# Turning around Cycles: An Approach Based on Selected Problems/Cases to Stimulate Collaborative Learning about Krebs and His Four Metabolic Cycles

**DOI:** 10.1021/acs.jchemed.1c01038

**Published:** 2022-05-25

**Authors:** Miguel Ángel Medina, Ángel Luis García-Ponce, Ángel Blanco-López, Ana R. Quesada, José Luis Urdiales, Ignacio Fajardo, Fernanda Suárez, Francisco José Alonso-Carrión

**Affiliations:** † Department of Molecular Biology and Biochemistry, Universidad de Málaga, Andalucía Tech, 29016 Málaga, Spain; ‡ Department of Mathematics Education, Social Sciences Education, and Sciences Education, Universidad de Málaga, Andalucía Tech, 29016 Málaga, Spain

**Keywords:** Second-Year Undergraduate, Biochemistry, Collaborative Learning, Problem Solving, Bioenergetics, Metabolism

## Abstract

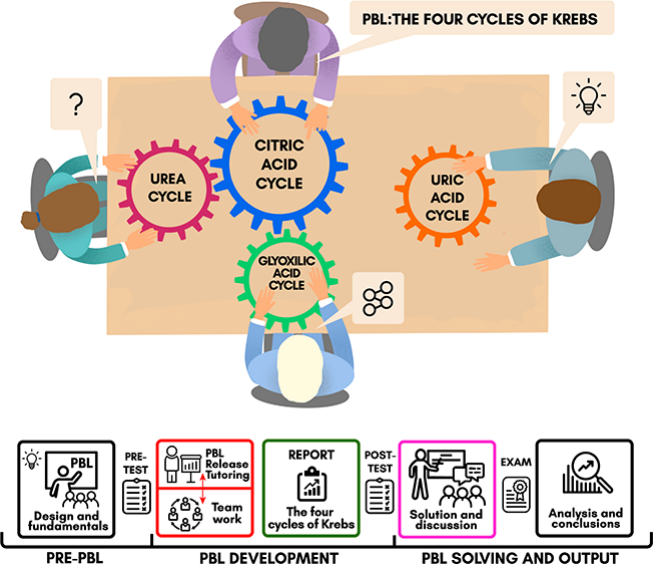

Metabolism is a challenging subject for bioscience students due to the intrinsic complexity of the metabolic network, as well as that of the overlapping mechanisms of metabolic regulation. Collaborative learning based on a problem-based learning approach can help students to successfully learn and understand metabolism. In the present article, we propose a selection of exercises, problems, and cases aimed to focus students’ attention on the scientific work made by Sir Hans Krebs and his collaborators to elucidate four main metabolic cycles, as well as on the study of these cycles, their regulation, and their metabolic integration. The objectives, the tools, and the implementation of this proposal are described, and the results obtained during its first implementation with volunteer students enrolled in two courses on metabolic regulation at our university are presented and discussed. These volunteer students signed a learning contract and were randomly distributed in small groups (3–4 students each). Application of this collaborative learning activity to our classrooms has been very satisfactory, as evidenced by an improvement in the volunteers’ academic performance and a very positive perception by most of them, who declared to be “very satisfied” or “satisfied” with their experience and felt that they had learned more.

## Introduction

The citric acid cycle is at the core of oxidative energetic metabolism. This cyclic metabolic pathway consists of eight enzymatically catalyzed reactions allowing for the rupture of the C–C bond of acetate with the concomitant production of both actual chemical energy (either ATP or GTP) and potential chemical energy (in the form of the reducing potential of NADH and FDAH_2_). In each turn of the cycle, two C atoms enter in the form of an acetyl-CoA and two C atoms (different from those entering in the same turn) are released in the form of two CO_2_ molecules, while one GTP (or ATP), 3 NADH, and one FADH_2_ are yielded. Furthermore, this central nucleus of oxidative metabolism is a metabolic roundabout to which main avenues of carbohydrate, lipid, and nitrogen metabolism converge and diverge. All this justifies why most biochemistry textbooks dedicate a separate chapter to explain this cycle, each of its enzymes, and its regulation.^1−3^

Very frequently, the citric acid cycle is called the Krebs cycle, in homage to Sir Hans Krebs, one of the most relevant biochemists of the 20th century and a Nobel prize winner, who played a key role in the elucidation of this metabolic pathway as a cycle.^4^ In contrast, it is not so well known that Krebs’ group contributed to the elucidation of three other metabolic cycles: the urea and the uric acid cycles, two pathways of nitrogen organic compounds metabolism, and the glyoxylate cycle, an anabolic pathway connecting lipids and carbohydrates in germinating oilseeds.^5,6^

In the present work, we described the design and contents of a teaching-learning sequence (TLS) consisting in a selection of exercises, problems, and cases regarding the scientific figure of Sir Hans Krebs and his four metabolic cycles, their regulation, and evolutive considerations as a useful tool to promote collaborative learning among undergraduate biochemistry students. We also analyze the impact of this activity on enrolled students.

## Objectives

With this experience, we aimed to achieve a main objective: To improve the teaching-learning process within courses on metabolism. Metabolism is a dynamic and complex network able to reprogram itself continuously in response to internal and environmental changes.^7^ This, along with its multiple levels of regulation and integration, contributes to make metabolism a study subject particularly difficult for biology, biochemistry, and other biosciences students.^8,9^ To achieve this first goal, and following the scheme of a complete TLS, a Problem-Based Learning (PBL) approach has been applied.^10−13^ Herein we will use the term PBL in its “weak” meaning, that is, learning based in the resolution of problems and cases, as it has been frequently used in articles published in journals of science education, including the *Journal of Chemical Education*.^14−21^ The active learning strategy presented in this manuscript was established by proposing a numerous and varied set of guided tasks, differing from the traditional PBL approach, in which a “problem” is presented for students to solve. The design, implementation, and evaluation of this teaching resource has been monitored through the use of an educational research methodology named Design-Based Research (DBR).^22^

A second objective was to contribute to change certain attitudes of students, decreasing their competitiveness and increasing their motivation and predisposition to collaborate. To achieve this goal, we stimulated students’ engagement with collaborative study procedures in a flipped classroom, thus allowing for a less hierarchical and more horizontal class, with the professor in the role of a facilitator/guide.^23^

## The Tools

### The Educational Research Methodology

As mentioned above, we made use of a DBR methodology.^24−26^ The term “design research” encompasses a broad spectrum of research approaches, basically of a qualitative nature, which share characteristic ways of approaching problems and the design of innovative educational environments. The purpose of design studies is to analyze, in order to understand and improve, the teaching and learning processes in specific contexts by systematically designing and studying particular forms of learning, teaching strategies, and tools.

**Figure 1:**
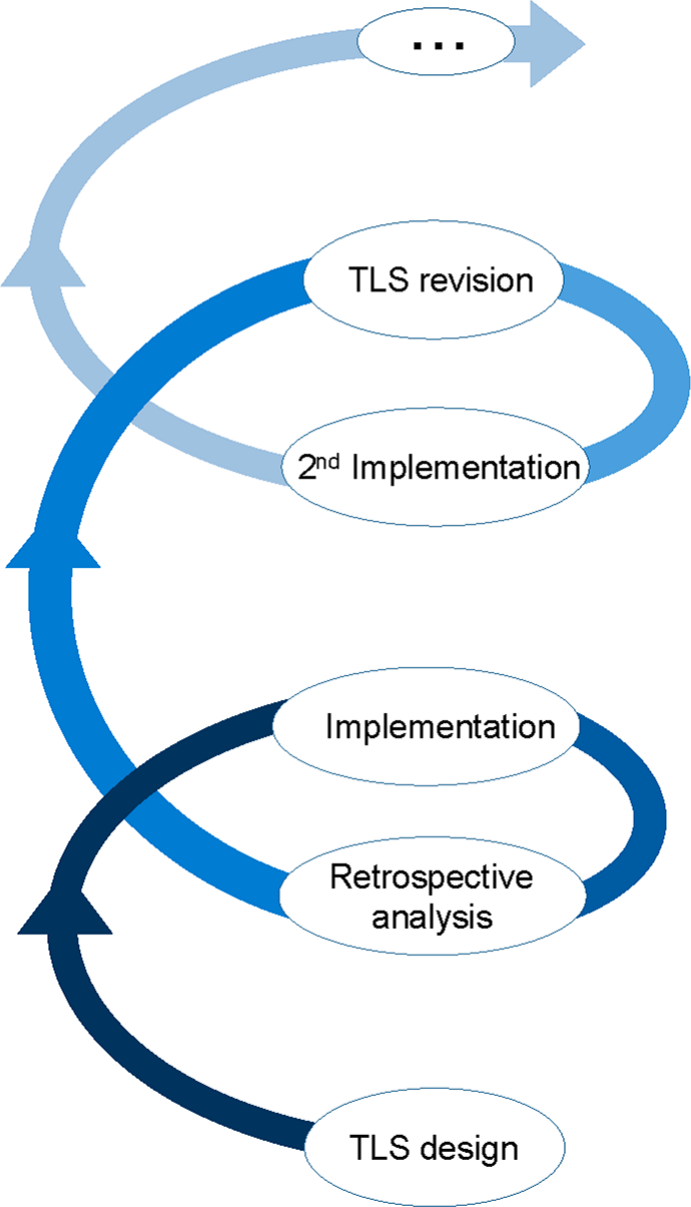
A simple scheme of the iterative Design-Based Research (DBR) methodology used to improve the Teaching-Learning Sequence (TLS).

This methodology makes use of a helix-shaped sequence of three iterative stages: (a) the stage of teaching resources design; (b) the stage of implementation of the design in the classroom; and (c) the stage of retrospective analysis, leading to potential improvements in the design stage in a second round.^27^Figure 1 is a scheme of the used DBR methodology. In the present study, we describe and discuss the first cycle of design, implementation, and retrospective analysis of the designed PBL.

### The PBL Design and Contents

During the academic course 2018–2019, we designed the contents of the PBL devoted to the four metabolic cycles described by Krebs and his group. It included 46 guided tasks organized around 4 topics, as shown in Table 1. The complete set of proposed exercises, problems, and cases, some of them with closed answers and others with open answers, covers different parts of the contents of a Metabolic Regulation course, including energy metabolism, anabolic conversion of lipids into carbohydrates, and nitrogen metabolism and excretion. The guided inquiries contained in the whole activity contribute to the commitment of small groups of students to be actively involved in a collaborative learning environment. An English version of the PBL is included here as Supporting Information SI1.

**Table 1:** Topics Covered by Our Proposed Problem-Based Learning (PBL) Case on the Four Metabolic Cycles Described by Hans Krebs

Topics covered	Number of guide tasks per topic
Historical issues regarding the scientific work made by Sir Hans Krebs	6
On the structure and properties of some molecules involved in Krebs’ cycles and the topology of these cycles	10
On the four metabolic cycles described by Krebs, their regulation, and their metabolic integration	21
On diseases linked to a bad functioning of the Krebs’ cycles	9

Some of the guided tasks were selected from popular biochemistry textbooks and students’ guides. Other tasks were designed with the aim of encouraging our students to read scientific articles or to make use of interesting online resources and biological databases. We consider particularly stimulating for our students the inclusion of tools and products generated and worked out by those of previous academic courses. Regarding this point, we should mention the project “The Krebs bicycle”, a radio program recorded and later presented in false direct by students of the subject Metabolic Biochemistry at the Botanic Garden of the University of Málaga (Spain) on June 1, 2013. The whole transcripts of this excellent, funny, and rigorous student-driven project are contained in a monographic issue of the popular science journal *Encuentros en la Biología* (Encounters in Biology, in Spanish), one of the published references we provide our students to read.^28^

Tables 2 and 3 list the articles and online resources, respectively, that our students had to consult to fulfill the tasks included in the PBL on the four metabolic cycles of Krebs.

**Table 2:** List of Articles That Students Had to Consult to Fulfill the Tasks Included in the PBL on the Four Metabolic Cycles of Krebs

Topics covered	Articles
A short story on α-ketoglutarate	Bootland^33^
Kpath tool	Navas-Delgado et al.^34^
Krebs and his trinity of cycles	Kornberg^5^
On a primordial, reversible Krebs cycle in a facultative thermophilic, chemo/lithosautotrophic organism	Nunoura et al.^35^
On a putative evolutive, nonenzymatic precursor of the Krebs cycle	Keller et al.^36^
On a putative reverse Krebs cycle under anaerobic conditions	Maden^37^
On adaptive responses to oxygen limitation in *E. coli*	Spiro and Guest^38^
On amino acid transporters in diseases	Bröer and Palacín^39^
On flux modes in the plant Krebs cycle	Sweetlove et al.^40^
On metabolic reprogramming	Medina^7^
On oncometabolites	Collins et al.^41^
On PubChem 3D viewer tool	Bolton et al.^42^
On Sir Hans Krebs’ biography and scientific achievements	Quesada^43^
On the forgotten fourth metabolic cycles described by Krebs	Salway^6^
On the reaction of fluorcitrate with aconitase	Lauble et al.^44^
PhenUMA tool	Rodríguez-López et al.^45^
The transcript of “The Krebs bicycle” and more	Pineda et al.^28^

**Table 3:** List of Online Resources and Biological Databases That Students Had to Consult to Fulfill the Tasks Included in the PBL on the Four Metabolic Cycles of Krebs

Online resources and biological databases	Topic covered
nobelprize.org	Nobel prize (1953) to Hans Krebs and Fritz Lipmann
wikipathways.org	Tool on metabolic pathways
browser.kpath.khaos.uma.es	Kpath tool (for metabolic pathways)
ebi.ac.uk/pdbe/	Database on protein 3D structures
pubchem.ncbi.nlm.nih.gov	Chemical compounds database
genecards.org	A human gene database
https://riuma.uma.es/xmlui/handle/10630/19824	A freely available book at the institutional repository of the University of Málaga
omim.org	A database on Mendelian inheritance in man
orpha.net	The European database of rare diseases
phenuma.clinbioinfosspa.es	PhenUMA tool (for phenotypes)
https://www.clinicaltrials.gov	A US database on clinical trials

### The Teaching-Learning Sequence and Its Implementation

**Figure 2:**
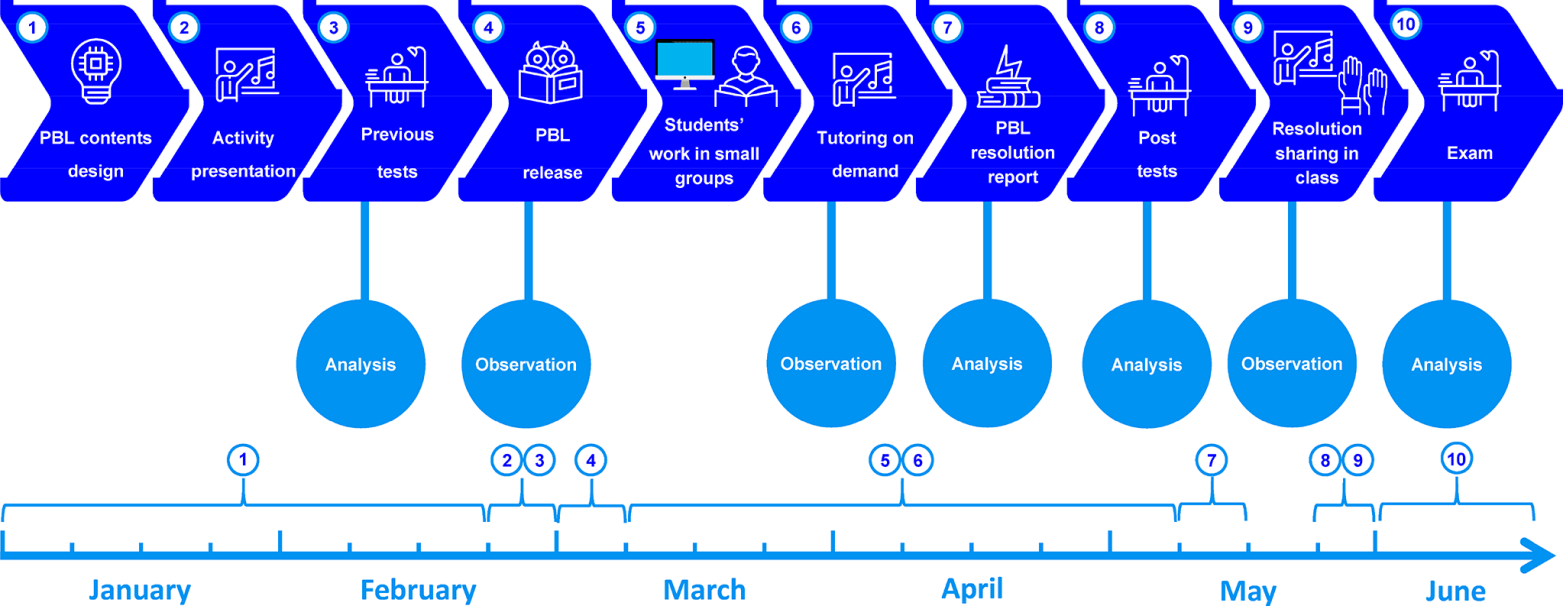
A simple flowchart of the TLS used in the present study, including a timeline.

Once the content of the PBL was established, we designed the TLS^29^ represented as a flowchart in Figure 2. This TLS was constituted by a series of sequenced teaching/learning activities, which we tried to adapt to the reasoning ability and mean knowledge of our students. In the application of the PBL to this research, the guided tasks were given in the form of step-by-step questions, and the students were directed to work cooperatively in groups via these guided tasks. In addition, web pages and resources were provided for the research subjects. The circles in Figure 2 indicate the different data collection instruments used in the research and the specific times at which they were used. Figure 2 also includes a timeline identifying the approximate moments at which the different steps of the TLS were carried out. The timeline has an arrow at the right indicating that the analysis of data continued being performed after the end of the semester.

Once the specific contents were designed and selected (1 in Figure 2), the TLS flowchart continued with the overall presentation of the activity to the students (2 in Figure 2), who took a test on previous knowledge (3 in Figure 2) on the four metabolic cycles described by Krebs (an English version is included as Supporting Information SI2) and a test on previous knowledge (3 in Figure 2) regarding the PBL methodology (an English version is included as Supporting Information SI3). After the tests, volunteer students were selected and randomly grouped, and PBL was released (4 in Figure 2). Each group of students worked independently from each other and freely decided how to share their tasks and how to organize their collaborative work. Groups had up to 2 months to prepare their final report (5 in Figure 2), containing a complete reasoned response for each task, as well as a public declaration of the contribution of each member of the group to the final report. Up to the deadline for the delivery of the resolution reports (7 in Figure 2), each group was allowed to demand guidance and tutorial sessions from their professors (6 in Figure 2). After this deadline, a special 3–4 h resolution sharing session was carried out at the classroom with the presence and participation of all the enrolled students (8 and 9 in Figure 2). At the beginning of this session, students repeated the tests on knowledge regarding the studied metabolic cycles and the PBL methodology. At the end of this session, students took a test on their perception regarding the activity and provide anonymously their overall rating of their degree of satisfaction with the activity (an English version is included as Supporting Information SI4). At the end of the season the final exam was presented to both enrolled and not enrolled students (10 in Figure 2). At the stages of PBL release, tutorial sessions on demand, and resolution sharing in class, professors and an external observer made and annotated their own observations. Tests, reports, and exams were subjected to analysis beyond the end of the semester in June.

### The Context of Implementation

Metabolism is a topic covered at the Faculty of Sciences of the University of Málaga (Spain) in 3 mandatory courses: one in the third academic year of the Degree in Chemistry, another one in the second academic year of the Degree in Biochemistry, and the third one in the second academic year of the Degree in Biology. These courses are mainly devoted to the study of metabolism, its regulation and its integration. This PBL was first implemented in the second semester of the academic course 2018–19 recruiting volunteer students enrolled in the courses of the Degrees in Biology and Biochemistry. Volunteer students signed a learning contract^30^ and were split in groups of 3–4 components chosen at random. Thirteen Metabolic Regulation (from the Degree in Biology) students were split in 4 groups and 47 Regulation of Metabolism (from the Degree in Biochemistry) students were split in 12 groups. The work with these students followed the TLS shown in Figure 1 and above commented.

In this work, the facilitator (M.A.M.), a full professor of Biochemistry and Molecular Biology with more than 30 years of teaching experience in these subjects, in addition to designing the activity, together with the other members of the research team (the authors of the article), presented the problem and the bibliographic resources to the students, proposed the work dynamics, attended the tutorials at the students’ request, directed the resolution sharing session, evaluated the PBL resolution reports, and designed and administered the exam.

## Results and Discussion

### Increased Capabilities of Enrolled Students in the Reading of Scientific Information

Most of our students arrive to the second-year course on metabolism with a poor or even nonexistent previous use of primary (original) or secondary (reviews) scientific literature. In fact, most of them declared that their own notes from the lessons were their primary source of information to prepare their courses and exams, followed by the eventual use of textbooks. Furthermore, most of our students were also not familiar with the use of biological databases and online resources. Actually, most of the URLs listed in Table 3 were previously unknown for our students. Therefore, to gain capabilities in the reading of primary and secondary scientific sources and to get familiarized themselves with those online resources are two major gains our volunteer enrolled students could obtain from this teaching experience.

### Knowledge Acquisition

**Figure 3:**
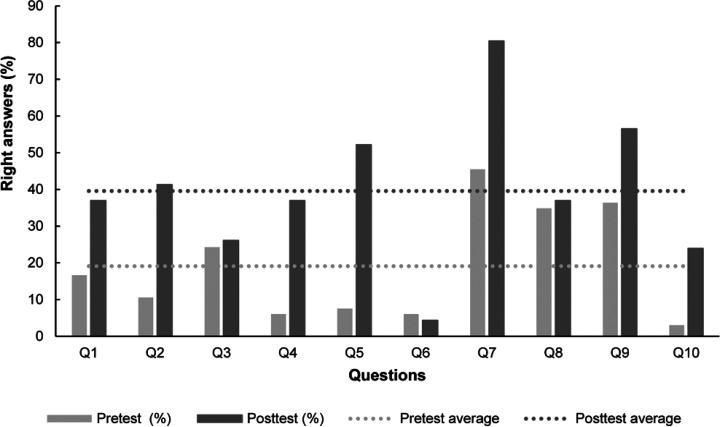
Percentages of right answers for each of the 10 questions included in the pre- and posttest of knowledge. The questionnaire is available (translated into English) as Supporting Information SI2.

Pre and post multiple-choice tests showed a relevant specific knowledge improvement on the Krebs’ cycles case. Figure 3 shows that in the most crowded course (that of undergraduate students in the Degree in Biochemistry) there was an increase in the percentage of right answers from a 19% in the pretest to a 40% in the posttest. Similar figures were obtained in the course corresponding to the Degree in Biology. These figures suggest that the whole activity had an overall positive impact on the knowledge acquisition by the enrolled students. As previously uncovered by others, from our experience it becomes evident that working cooperatively in small groups on a complex collection of problems and cases has a very positive and robust effect on students learning biochemistry.^31^ However, there is still ample room for improvement. On the one hand the overall percentage of right answers in the posttest is still low. On the other hand, for questions Q3, Q6, and Q8 there was no improvement at all. This is in contrast with the overall high scores that most of the groups obtained with their reports. A brief selection of responses provided by students’ groups in their reports is included as Supporting Information SI5.

PBL methodology had a real impact not only in the overall knowledge of Krebs’ metabolic cyclic pathways but also on the study of the course on metabolic regulation as a whole for most of the enrolled students in both groups of the Biochemistry and Biology Degrees. In Metabolic Regulation (from the Degree in Biology) only 16% of students that did not enroll in this volunteer activity passed the final exam, in high contrast with the 46% of enrolled students that passed the final exam. This difference was even greater in Regulation of Metabolism (from the Degree in Biochemistry), where 20% of not enrolled students and 77% of enrolled students passed the final exam.

### Students’ Previous Knowledge about the Methodology and Final Perception about the Activity

**Figure 4:**
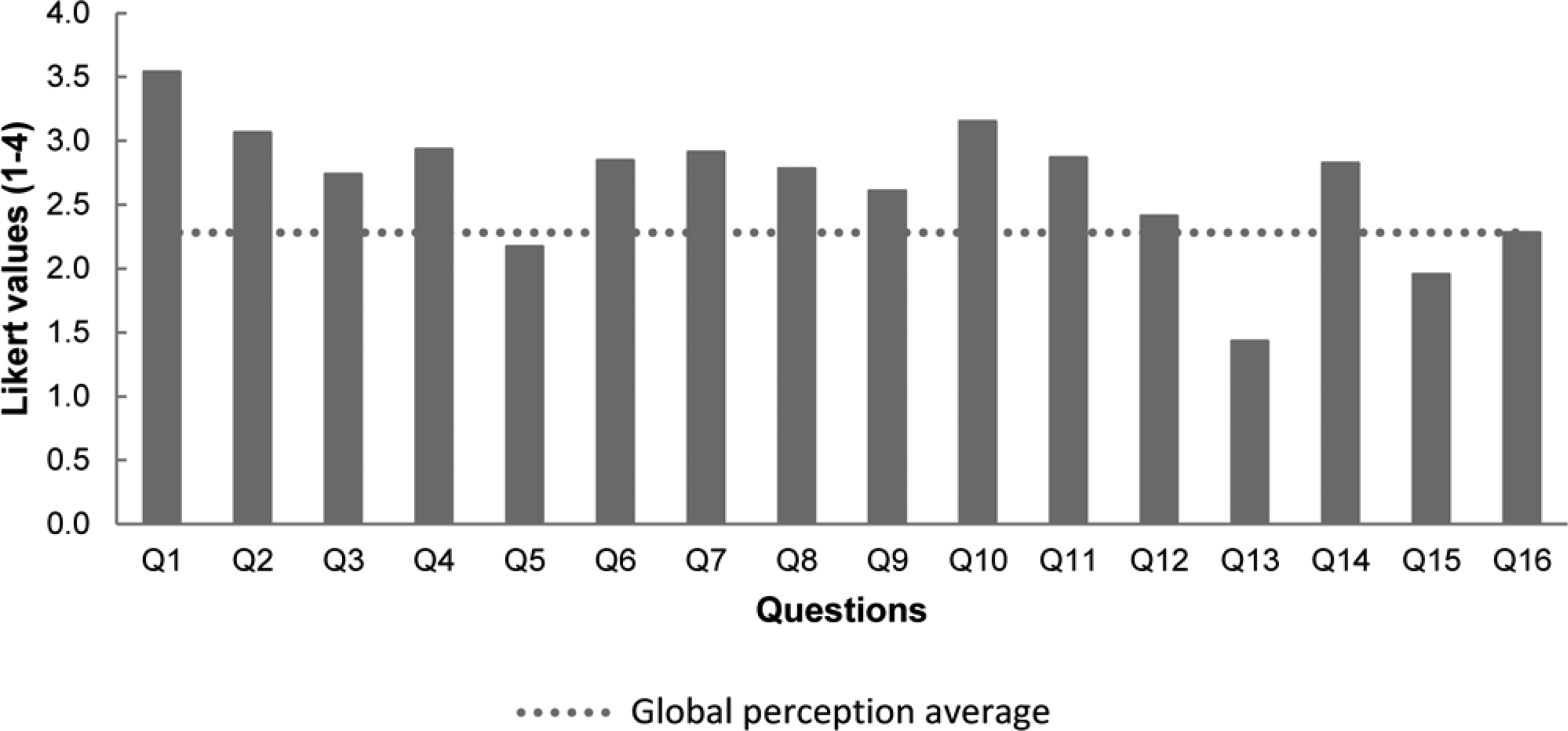
Results on the enrolled biochemistry students’ perception test consisting of 16 closed questions, each with four Likert values (1 to 4, from less to more satisfied). The questionnaire is available (translated into English) as Supporting Information SI4.

Regarding the PBL problem solving methodology, most of the students (both enrolled and not enrolled to this activity) declared they have not heard of and have never used the PBL approach prior to this course (results obtained from the test included as Supporting Information SI3). On the other hand, there was a general student consensus that they improve their learning experience by using this type of PBL, despite the major effort and dedication to solve the case (see Figure 4). Q1 (“*I find this course useful and interesting*”) addressed an overall perception by students on the course, which was mostly positive. Q16 is the overall rate provided by students to the PBL activity, which was not higher mainly because students perceived that this methodology requires more work and preparation (Q13).

There was an open question (Q17) asking: “*After taking this course, what does Problem Based Learning mean to you?*” Some responses provided by students to this question are shown in Supporting Information SI6. Q18 contained nine brief statements that should be valued from 1 (minimum) to 4 (maximum) with regards to (i) the degree of experience acquired by the students and (ii) the importance that the student attaches to each of the statements. The overall score for both was high, and very close to the maximum value 4 in the second case.

## Conclusion

Our first experience with the application of the PBL approach to general course on metabolic regulation was carried out in the academic course 2017–2018 with a PBL case devoted to the study of glycogen, its metabolism, its regulation, and its integration.^17^ The tricarboxylic acid cycle is central for energy metabolism and a key topic to understand metabolic regulation. Although this Krebs cycle has been the center of the contents of some ten previous articles in the *Journal of Chemical Education*, none of them had a comprehensive approach such as this contribution describing the use of a collection of problems and cases within a TLS designed to have a real impact on students’ learning of metabolism. On the other hand, the other three Krebs’ cycles had no previous presence in the journal. Furthermore, a search within the ERIC database revealed that there was no previous article published in education and science education journals using this kind of approach for the teaching of Krebs metabolic cycles. Therefore, the present article contributes to full this gap in the literature.

The results obtained with the first implementation of the present PBL devoted to the four metabolic cycles confirms our initial conclusions: The increase in the percentages of correct answers in the posttest as compared with the pretest was greater among the volunteers than among the students that did not sign the learning contract. Furthermore, global scores were remarkably better for those volunteer students that had signed the learning contract rather than for those who did not. The contribution to these better scores of a relatively higher interest in the subject by enrolled students cannot be discarded.^31^ Most of the volunteers declared that they felt that this PBL approach has been useful for them, believed that they had learned more, but that they also have worked more and harder than for the resolution of other kinds of tasks. Overall, around an 80% of students enrolled in this study declared to be “very satisfied” or “satisfied” with their experience. This overall positive perception is clearly connected with the fact that the PBL approach actually was motivating and promoted the collaboration among the enrolled students. As previously revealed by others, PBL has a direct impact on students’ skills, mainly on those allowing them to work in teams successfully.^31,32^ Nonetheless, this study has still a major limitation, due to the relatively small number of enrolled students in this volunteer experience. Another important limitation is that this activity focuses only on some parts of the extensive contents of the syllabus of the metabolic regulation courses. During the four academic courses since the beginning of this kind of activity, the cumulative number of enrolled students has increased gradually, allowing us to reinforce our main conclusions. In these years, the second and subsequent cycles of TLS design within the framework of DBR methodology (Figure 1) have led us to introduce two slight but important changes in the TLS shown in Figure 2, namely the following: (1) During the two months where students work in small groups, we have added three compulsory progress sessions coordinated by the professor/facilitator in which all the enrolled groups exchange their experience and anticipate their respective progress in the activity. (2) The PBL activity is not released as a whole (point 4 in Figure 2) but in four parts, one at the beginning of students’ work on the activity, and the next ones immediately after each of the three compulsory progress sessions. Currently we have a number of different PBL cases covering most of the total contents of our metabolic regulation courses.

## Supporting Information Available

SI1: English version of the proposed PBL (PDF)
SI2: English version of the test on students’ knowledge regarding the four metabolic cycles of Krebs (PDF)
SI3: English version of the test on students’ previous knowledge of the PBL methodology (PDF)
SI4: English version of the perception test on Krebs and his four metabolic cycles PBL (PDF)
SI5: Some selected responses by students to problems and cases included in the PBL (PDF)
SI6: Some responses to the open question Q17 in the Perception Test (PDF)
