# Supplementary material for: Turning around Cycles: An Approach Based on Selected Problems/Cases to Stimulate Collaborative Learning about Krebs and His Four Metabolic Cycles
Source: J Chem Educ. 2022 May 25;99(6):2270–6. doi: 10.1021/acs.jchemed.1c01038 (PMC9202563; doi:10.1021/acs.jchemed.1c01038)
Supplement: Supplementary file 1 — ed1c01038_si_001.pdf [file ed1c01038_si_001.pdf]

PBL (Problem or case-based learning)

Study of the Krebs cycles, their metabolism and regulation, and related diseases

To begin with. This type of task lends itself to the use of the ***collaborative learning*** method in small groups of students. However, there is always an unavoidable individual learning task. With this case we want to thoroughly review a "collateral" metabolic pathway dissected by many biochemical studies over more than 70 years of research. As a starting point, the components of the various groups should individually review what they had to study in Biochemistry I or Fundamentals of Biochemistry about the structure and physicochemical properties of glycogen. It is also highly recommended that they review the topic of glycogen metabolism and its regulation each in their favorite book of Biochemistry with the help of the "Guide to Contents" of the topic available in the Virtual Campus.

Basic rules of the work. 1. Each group will work autonomously on the case and decide on its work strategy, which should guarantee the highest degree of collaboration between the group members. The work strategy and the justification of the degree of collaboration that it guarantees must be set out and commented on explicitly in the final report. 2. The components of the group should agree on the drafting of a single joint final report that will answer in detail (and always in a properly argued manner) all the questions that arise in the case. 3. The report should include an explicit statement of the degree of participation and specific contributions of each component of the group to the resolution of the case. 4. The following structure is recommended for the report: a) Cover with the title of the work and the composition of the group. b) Table of contents. c) Declaration of the degree of participation and the specific contributions of each component of the group. d) Body of work: Detailed and reasoned resolution of all the questions proposed in the case. e) Explicit declaration of all the bibliographical references, URLs, tools and utilities used in the resolution of the case. You must take care that the citations are complete, correct and that the same system of citation is used consistently. f) The group that wishes can add a final section proposing new questions and exercises around the same case, notifying the sources used. 5. The report must be submitted in the specified form by the teacher. 6. Each group must guarantee that all its members have understood and assimilated the answers given by the group to all the proposed questions and exercises. In this way, in the case of a public and face-to-face sharing (or in any other way in which the degree of learning of the components is evaluated) each component of the group could be responsible for explaining all the answers given by the group. 7. Throughout the process, the various working groups may request guidance and mentoring from the teacher when they feel it is necessary.

1. Historical aspects of the scientific studies carried out by Sir Hans Krebs

1.1. Mar Quesada Molina, student of the subject Metabolic Biochemistry of the former Bachelor's Degree in Biology in the academic year 2011-2012 wrote as a voluntary work for that subject the text "Hans Krebs and the discovery of the cycle that bears his name", which was later included in the monographic issue of the journal Encounters in Biology dedicated to the experience "Krebs' bicycle" (**Quesada. Encounters in Biology 7 (148): 85-93**). i) Read this text and from it elaborate a summary table with the most important milestones. ii) List all the scientists mentioned in the text. iii) Investigate their respective scientific legacies and elaborate a brief "biographical dictionary" in which, in alphabetical order of surnames, you dedicate an entry to each one of them, highlighting the main data of their scientific lives.

1.2. Krebs shared the Nobel Prize in Physiology or Medicine with one of the other scientists mentioned in Mar Quesada's work. Consult the official website of the Nobel Prize (URL: nobelprize.org) to identify the year in which this prize was awarded and the justification used by the committee in the official announcement of the Nobel Prize.

1.3. In the same Nobel Prize web site you can access the presentation of the prize made at the award ceremony by Professor E. Hammarsten. Based on the information provided by this presentation, write a brief summary of the scientific merits of the winners.

1.4. You can also access the Nobel Lectures given by the two laureates. i) From Krebs' lecture, comment and explain the information provided by Table 1 and that provided by Figure 2. ii) In Krebs' lecture, an article by Severo Ochoa is mentioned. Identify it, look it up, put its full citation and add in an appendix the article by Ochoa downloaded from its source. Why does Krebs mention this article? What does this article contribute to the history of the Krebs cycle? iii) Explain the results shown in the Tables and Figures of the Nobel Lecture given by the other awardee. Summarize in less than 600 words the contents of this second lecture.

1.5. On the occasion of the first centenary of the birth of Hans Krebs, H.L. Kornberg wrote an article entitled "*Krebs and his trinity of cycles*" (**Kornberg HL. Nat Rev Mol Cell Biol 1: 225-228, 2000**). In this short text, Kornberg reviews three of the metabolic cycles that Krebs characterized. However, Jack Salway in a brief letter published in November 2018 in TIBS (**Salway JG. Trends Biochem Sci 43: 847-849**) remarks that Kornberg forgot to mention a fourth cycle described by Krebs. Download Salway's article and search for and download references 2 through 5 of the article. With this information, prepare an essay summarizing how Krebs and his collaborators describe each of the four Krebs cycles.

1.6. Look up in the course content guides the context in which the different Krebs cycles are explained. With all the information handled, elaborate a brief essay on the contributions of the Krebs laboratory to the current knowledge of primary metabolism.

2. On the structure and properties of some molecules involved in Krebs' cycles and on the toplogy of these cycles.

2.1. A very useful tool for the study of metabolic pathways is ***Wikipathways*** (URL: wikipathways.org). Search *Wikipathways* for the map for each of the three first Krebs' cycles, download them, insert them in your report and comment them.

2.2. Another very useful tool for the study and comparison of metabolic pathways is ***kpath***. The article **Navas-Delgado et al., Database 2015, 1-11** explains the functionality and use of this freely available tool (URL: browser.kpath.khaos.uma.es). Explore with *kpath* the metabolic pathways mentioned in 3.1,, download the metabolic maps provided by the tool and comment on the similarities and differences with the maps obtained with *Wikipathways*.

2.3. Download diagrams of the three cycles mentioned in 2.1 from Lehninger's Principles of Biochemistry, include them in your report and comment on the differences found with the maps obtained in 2.1 and 2.2.

2.4. Download the map of the uric acid cycle from the Salway reference cited in 1.5, include it in your report and comment on it.

2.5. ***PDB*** is a freely accessible database that provides information on the three-dimensional structure of proteins. You can access the European *PDB* portal at the URL: ebi.ac.uk/pdbe. Search in *PDB* some image of the crystalline structures of citrate synthase, malate synthase and arginine succuniate lyase. Include in your report the downloaded images, their respective PDB references and a brief comment to each one.

2.6. A very useful tool to understand in more detail the structure and properties of chemical compounds is ***PubChem*** (URL: pubchem.ncbi.nlm.nih.gov). Search in *PubChem* for the information it contains about the structure and properties of acetyl-CoA, glyoxylate and urea, and extract the most relevant information to include in your report.

2.7. *PubChem* uses the ***PubChem3D*** viewer to generate 3D models of formulas for most of the chemical compounds in the *PubChem* database. The article **Bolto et al. J Cheminformatics 3: 32 (2011**) describes what *PubChem3D* is and how it works. It is recommended that you consult it before using the tool to generate your own representations of the spatial conformation of citrate, acetyl-CoA, glyoxylate, arginine succinate and urea. A selection of the images generated by you with the tool should be included in your report with a brief description of what each image shows.

2.8. Look up in the manual "***Biochemistry, 4th ed***" by Voet and Voet (2011) problems 1, 7 and 9 of its chapter 21, devoted to the Krebs cycle. Translate into English the statements of the three problems and propose the solutions of 1 and 7, explained step by step. Problem 9 is based on the information contained in the article by **Lauble et al. Proc Natl Acad Sci USA 93: 13699-13703, 1996**. Download the article and use its information to answer problem 9 in a reasoned manner.

2.9. A bicycle is made from two wheels. Look in **Stryer's biochemistry textbook** for a schematic of the Krebs cycle, download it, include it in your report, and discuss its highlights.

2.10. ***The Krebs bicycle*** was an initiative encouraged by the five students that make up the "Krebs bicycle" team (Hugo Pineda, Lola Nevado, Sara Bernárdez, María Jesús Pacheco, Juan Jesús Criado and Florencio Palomas) and that achieved the active participation of more than half of the students enrolled in the 2012-13 academic year in the Metabolic Biochemistry course of the former Bachelor's Degree in Biology. In the words of the promoters of this initiative: "Who hasn't thought at some point: 'What a boring class'? Couldn't there be a way to learn this in a more enjoyable and fun way? Although we are sometimes led to believe that there isn't, there is". Krebs' bicycle is a good example that there are positive answers to this last question. An answer, moreover, that in this case has been found, created, developed and carried out by the students themselves in the format of a radio program. The first monographic issue of ***Encounters in Biology*** of the year 2014 collects this happy student initiative, including the transcription of all the contents of the radio magazine that had its "world premiere" at the Botanical Garden of the University of Malaga on Saturday, June 1, 2013. Download this monographic issue of Encounters in Biology from the Virtual Campus of the subject, read its contents and use the links if you want to listen to them. Finally, write an essay commenting on the highlights of the experience.

3. On the four metabolic Krebs cycles, their regulation and their metabolic integration.

3.1. A simple way to have a first approximation to the regulation of a metabolic pathway is to critically analyze a table with the values of free energy variations of the different metabolic steps. Download the table with this information corresponding to the steps of the Krebs cycle that can be found in Stryer's Biochemistry manual. Copy and include this table in your report. From your information, what can you deduce about the regulation of the cycle and, specifically,

which catalytic steps can be inferred to play the most important role in its regulation? Find out and comment on the most salient aspects of the three key enzymes of the cycle.

3.2. Several metabolic regulatory mechanisms discussed in topic 1 are involved in the regulation of Krebs cycles. i) What is meant by regulation by "metabolic control" and what role does this regulatory mechanism play in the control of the Krebs cycle? ii) What is meant by "metabolic channeling" and what role does it play in the regulation of the Krebs cycle? Find an original research article that described the "metabolon" of the Krebs cycle. Include the full citation in your report and comment on the contents of the article. Define "metabolon" and find who originally proposed the concept. iii) What is regulation by "metabolic compartmentation"? What role does metabolic compartmentation play in the co-regulation of the Krebs cycle and the glyoxylate cycle in oilseeds?

3.3. The urea cycle is a compartmentalized pathway between cytoplasm and mitochondria. This implies the necessary involvement of inner mitochondrial membrane transporter systems in the process. However, the maps of the cycle depicted in biochemistry textbooks do not emphasize this fact. i) Find which transporter systems are integral to the urea cycle. Download Figure 4 from the review by **Bröet and Palacín. Biochem J 436: 193-211, 2011** and comment on it, highlighting the central role of the ORC transporter system and the collateral role of AGC, OGC and CIC in the urea cycle. ii) Search PDB and GeneCards (https://www.genecards.org) for information on ORC structure and function.

3.4. Investigate the dual role of the amino acid arginine as a cytoplasmic substrate and mitochondrial regulator of the urea cycle.

3.5. In the contents guide of the topic corresponding to the metabolism of nitrogen compounds you can find some diagrams extracted from chapter 27 of the first edition of the ***Biochemistry textbook by Emilio Herrera*** that help to draw an evolutionary scenario for the urea cycle. Download these diagrams, include them in the report and write an essay based on them about the evolutionary origin of the urea cycle in humans.

3.6. In the same content guide you can find information on the relationship of the urea cycle with the intercellular glutamine synthetase/glutaminase cycle. i) Describe this cycle and indicate three systems in the human organism in which this cycle occurs. ii) Explain how the interaction of the urea cycle and this cycle contribute to maintaining blood pH homeostasis and why this is physiologically relevant.

3.7. From the analysis of the Stryer table mentioned in 3.1 an interesting speculation on the evolutionary origin of the cycle can be made. i) What is the standard free energy value of the reaction catalyzed by the enzyme malate dehydrogenase (MDH)? If the reaction were to occur in isolation under these conditions, would it be spontaneous? How do you explain that MDH functions in the malate to oxaloacetate sense in the Krebs cycle? ii) A few years ago Biochemistry II students involved in some intensified continuous assessment activities designed T-shirts claiming ***"We are all malate dehydrogenases!"*** Comment on the meaning of this expression and this initiative in the context of the study of the regulation of metabolism. iii) What would happen to the Krebs cycle if the 2-oxoglutarate dehydrogenase complex did not work? Calculate the overall free energy variation of each of the two functional branches of the Krebs cycle under these conditions.

3.8. In facultative anaerobic organisms the "complete" Krebs cycle functions under aerobic conditions, whereas in anaerobiosis the cycle becomes a branched biosynthetic pathway. On page 18 of the book "***Metabolic Reprogramming as a Target for Cancer and Other Diseases***", downloadable from the institutional repository of the UMA (https://riuma.uma.es/xmlui/) you can find a figure that illustrates the reprogramming of energy metabolism in yeast. i) Download the figure, include it in the report and comment on it. ii) Write down the complete citation of the article by **Mayor Zaragoza**'s group that demonstrated this in the first place. Comment on the contents of that article. iii) In chapter 21 of the **fourth edition of**

Voet and Voet's Biochemistry textbook you can find a section on the evolution of the cycle. Read it and summarize its salient information. iv) Spiro and Guest (Trends Biochem Sci 16: 310-314, 1991) review what was known at that time about adaptive responses to oxygen limitation in another facultative organism, the enterobacterium *E. coli*. Look up the article, include it in your report, and comment on the information gleaned from its figures.

3.9. The different modes of flow through all or part of the metabolic steps of the Krebs cycle are a paradigmatic example of the flexibility and adaptability of metabolism to different physiological and environmental conditions. The classic review by Sweetlove et al. (**Trends Plant Sci 15: 462-470, 2010**) on the modes of operation of the cycle in plants superbly illustrates this fact. Look up the article, copy its 5 figures and comment on the information contained in each figure.

3.10 Duncan Bootland wrote an essay for a biochemistry course by Professor D.A. Bender in story format. Professor Bender liked it so much that he sent it for publication with his student's authorship in the journal ***Biochemical Education (26. 14-15, 1998***). i) Find and read the story, include your English translation in your report, and comment on it. ii) Dare to write a story with the title "We are all malate dehydrogenase!" (mentioned in 3.7).

3.11. Wachtershäuser has proposed several theoretical scenarios for a prebiotic metabolism and origin of life in the vicinity of oceanic volcanic hot springs. In particular, he has proposed a kind of reverse Krebs cycle under anaerobic conditions in which ferrous sulfide would react with hydrogen sulfide to generate FeS2 and give off hydrogen gas. You may find the summary of Wachterhäuser's proposals in Maden's article in **Trends Biochem Sci 20: 337-341, 1995**, helpful as a guide. With this information, propose how the reverse Krebs cycle would work under these conditions.

3.12. In 2017, Keller et al published an article suggesting a possible non-enzymatic evolutionary precursor of the Krebs cycle (**Nat Ecol Evol 1: 0083, 2017**). Find the article, read it and summarize it by commenting on its 4 figures.

3.13. In 2018, Nunoura et al published an article in Science describing a primordial and reversible Krebs cycle in a facultative chemolithoautotrophic thermophile (**Science 359: 559-563, 2018).** i) What do "***thermophile***" and "***chemolithoautotroph***" mean? ii) Download the article and copy into your report the 3 tables and 2 figures from the main text of this article. Comment on each of them, highlighting the key information extracted from each.

3.14. In the fifth edition of ***Lehninger Principles of Biochemistry***, problem 36 of the chapter on the citric acid cycle is entitled "*How the citric acid cycle was determined*". Copy the English translation of its statement and answer each of its 8 questions in detail.

3.15. i) The Krebs cycle is said to be an ***amphibolic*** metabolic pathway. Define this concept and justify why the Krebs cycle is and how. ii) Related to the Krebs cycle are the so-called ***anaplerotic*** reactions. Define them and identify the main anaplerotic reactions of the Krebs cycle, describing them.

3.16. i) Why can the Krebs cycle be interpreted as the "***central core of oxidative metabolism***"? ii) Why is it sometimes metaphorically compared to a traffic circle? iii) Why is the Krebs cycle first presented in the content guide of the topic dedicated to the central core of oxidative metabolism under the title "***the breaker cycle***"?

3.17. i) Fat catabolism produces a large amount of acetyl CoA, which primes the Krebs cycle. An intermediate of the cycle, oxaloacetate, is used as a starting point for glucose synthesis in gluconeogenesis. Why after strenuous exercise that has depleted our glycogen stores must these be replenished with carbohydrate intake? Why don't we replace the carbohydrates consumed in exercise by metabolically converting our fats to carbohydrates? ii) What effect does intense fatty acid oxidation have on the activity of the PDH enzyme complex and on glycolysis?

3.18. i) A cell is deficient in pyruvate dehydrogenase phosphate phosphatase activity. How will this deficiency affect cellular metabolism? ii) Another cell has a defect in the Krebs cycle

resulting in inhibition of PDK2. What effect does this have on the transcription factor HIF-1? Compare the effects of this situation with that of the previous case on cell metabolism.

3.19. Although oxygen is not directly involved in the Krebs cycle, the Krebs cycle only functions when oxygen is available. Why?

3.20 i) A person on a particular diet consumes 10000 kJ/day and excretes 40 g of urea. Assuming that protein contains 16% by weight of N and that its metabolism yields 18 kJ/g, what percentage of this person's energy intake is achieved by his protein metabolism? ii) The production of urea cycle enzymes increases or decreases as a function of metabolic needs. There are high levels of these enzymes in both protein-rich diets and under fasting conditions. Explain this apparent paradox.

3.21. Prepare an essay detailing the integration of fermentative and oxidative metabolism in a growing tumor.

4. Diseases linked to a bad functioning of the Krebs metabolic cycles. Biochemical substantiation of clinical cases.

4.1. Search Wikipathways for the metabolic map of the pathologies associated with Krebs cycle dysregulation, download it, include it in your report and comment on it.

4.2. a) What is an *inborn error of metabolism*? b) Find out and identify the author who first used this expression. c) To which metabolic disease did he apply this expression? d) What does this disease consist of, what are its biochemical bases and what are its clinical consequences?

4.3. ***OMIM*** (Online Mendelian Inheritance in Man) is an extensive online catalogue (URL: omim.org) of human genes and genetically based diseases, including inborn error of metabolism. On the other hand, the most important database portal for rare diseases is ***Orphanet*** (URL: orpha.net). a) Search for the OMIM and ORPHA codes corresponding to each of the diseases mentioned in 4.1. b) Download a summary sheet with the most important information associated with these diseases in OMIM and Orphanet. c) Summarize the differential features of DOOR syndrome.

4.4. ***PhenUMA*** is a web application that was designed, among other things, to help the study of rare diseases and their gene relationships. The article **Rodríguez-López et al., BMC Bioinformatics 15: 375 (2014)** describes PhenUMA and its applications. PhenUMA is currently hosted at the URL: phenuma.clinbioinfosspa.es. Make use of PhenUMA to further study diseases linked to defects of the urea cycle diseases and generate for each of them a network of functionally related genes.

4.5. i) Use OMIM, Orphanet or PhenUMA to find out if there is any disease associated with a defect in the functioning of any of the enzymes involved in the uric acid cycle. Comment your results. ii) Explain in detail the metabolic basis of gout and look for information on its etiology, symptomatology and treatment.

4.6. Alcohol is identified as a ***xenobiotic*** by our body and metabolized for inactivation. i) What are xenobiotic compounds? ii) In what organ and by what set of processes are they metabolized? iii) What enzyme activities metabolize alcohol? What properties do these enzymes have? iv) How is the NAD^+/NADH ratio affected by high intakes of alcohol? How is the Krebs cycle affected? v) What is the microsomal system of ethanol oxidation, in which cellular compartment does it take place and what are the metabolic consequences of an excess of this pathway? vi) What are liver transaminases, what are the normal ranges of their activities in a healthy person and how are these values likely to be in a chronic alcoholic?

4.7. i) Inquire what is meant by ***oncometabolite***. ii) Download the article by Collins et al (**Clin Chem 63: 1812-1820, 2017**), copy their Figure 1 about Krebs cycle oncometabolites and write an essay about the biological origin of each of them and their role in cancer.

4.8. The American Food and Drug Administration (**FDA**) recently approved the use of ***enasidenib*** for the treatment of oncology patients with mutations in the IDH2 gene. i) What is the FDA and what are its missions? ii) What type of compound is enasidenib, what target does it have, and what effects does it cause?

4.9. i) What is meant by a "***clinical trial***"? ii) What phases does each phase of a "clinical trial" consist of and what is its purpose? iii) The National Institutes of Health (NIH) portal https://www.clinicaltrials.gov is a database that collects clinical trial studies from all over the world. Search this portal for clinical trials directed against enzymes involved in oncometabolite production. Download the information obtained, include it in the report and comment on it.
