# Supplementary material for: Turning around Cycles: An Approach Based on Selected Problems/Cases to Stimulate Collaborative Learning about Krebs and His Four Metabolic Cycles
Source: J Chem Educ. 2022 May 25;99(6):2270–6. doi: 10.1021/acs.jchemed.1c01038 (PMC9202563; doi:10.1021/acs.jchemed.1c01038)
Supplement: Supplementary file 2 — ed1c01038_si_002.pdf [file ed1c01038_si_002.pdf]

“Nickname”: ________________

TEST (ANONYMOUS) OF PRIOR KNOWLEDGE

Before you start: Point out which group you belong to:

A) Students who have signed the "learning contract" ☐

B) Students who have NOT signed the "learning contract" ☐

C) First time students in the subject ☐

D) Students in second or successive registration in the subject ☐

Test questions. In each case, indicate next to the question number the letter corresponding to the ONLY correct option (these are questions with only one valid answer).

1. The Nobel Prize of Medicine or Physiology to the studies on glycogen metabolism was shared by how many scientists:

A) 1.

B) 2.

C) 3.

D) 4.

E) 5.

2. On the hormonal regulation of glycogen metabolism by insulin and glucagon

A) Both regulatory pathways are mediated by protein kinases A.

B) Both regulatory pathways are mediated by protein kinases B.

C) Regulation by insulin is mediated by protein kinases A and regulation by glucagon is mediated by protein kinases B.

D) Regulation by insulin is mediated by protein kinases B and regulation by glucagon is mediated by protein kinases A.

E) All of the above is false.

3. What glycogenosis ins caused by a bad functioning of the debranching enzyme:

A) Pompe disease.

B) Andersen disease.

C) Cori disease.

D) Hers disease.

E) Fanconi-Bickel disease.

4. How many metabolic cycles were elucidated by Hans Krebs’ group:

A) 1.

B) 2.

C) 3.

D) 4.

E) 5.

5. What other famous biochemist shared Nobel prize with Hans Krebs:

A) Albert Lehninger.

B) Otto Warburg.

C) Lubert Stryer.

D) Fritz Lipmann.

E) Severo Ochoa.

6. How many non-equilibrium reactions take part in the tricarboxylic acid cycle:

A) 1.

B) 2.

C) 3.

D) 4.

E) 5.

7. What is the most important mechanism of global regulation of the tricarboxylic acid cycle:

A) Metabolic compartmentation.

B) Gene expression regulation.

C) Metabolic control regulation.

D) Covalent modification.

E) Enzyme turnover.

8. It does not take part in the urea cycle:

A) PABC transporter.

B) Carbamoyl phosphate synthetase II.

C) Arginase.

D) Citrulline.

E) Ornithine transcarbamoylase.

9. It contributes to the maintenance of the blood pH homeostasis:

A) The carbonate/bicarbonate buffer.

B) The glutamine synthetase/glutaminase cycle,

C) The urea cycle.

(D) All the previous options are valid.

E) The glyoxylate cycle.

10. In which of the following metabolic diseases is involved a deficiency in the tricarboxylic acid cycle:

A) Citrullinemia.

B) Phenylketonuria.

C) von Gierke's disease.

D) von Hippel-Lindau syndrome.

E) DOOR sydrrome.

Very brief questions, which can be answered in one line.

B1. Mention at least two disciples of Hans Krebs.

____

B2. How many turns of the Krebs cycle are needed to release as carbon dioxide those C atoms that enter the cycle from fatty acid oxidation?

____

B3. Mention two anaplerotic reactions.

____

B4. What is the meaning of the expression «the breaker cycle» when applied to the Krebs cycle?

____

B5. How many enzyme activities shows the enzyme complex 2-oxoacid dehydrogenase and what cofactors take part in its functioning?

____

B6. What two different metabolic function does arginine paly in the urea cycle?

____

B7Identify at least one oncometabolite related with the Krebs cycle.

____
