# Supplementary material for: Turning around Cycles: An Approach Based on Selected Problems/Cases to Stimulate Collaborative Learning about Krebs and His Four Metabolic Cycles
Source: J Chem Educ. 2022 May 25;99(6):2270–6. doi: 10.1021/acs.jchemed.1c01038 (PMC9202563; doi:10.1021/acs.jchemed.1c01038)
Supplement: Supplementary file 3 — ed1c01038_si_003.pdf [file ed1c01038_si_003.pdf]

“Nickname”: ____________

QUESTIONS ABOUT "PROBLEM-BASED LEARNING" (PBL)

Circle the letter corresponding to the answer of your choice for each question.

1. Prior to this course, did you know or have you heard of Problem-Based Learning (PBL)?

a. Yes

b. No

2. Have you ever employed this methodology?

a. No

b. Yes

If so, briefly explain in what context you used it (educational level, subject, etc.)

____

____

____

____

Read and evaluate from 1 to 4 (1-minimum; 4-maximum) your level of experience and the importance you give to the following:

		Experience	Importance
**3.**	To solve a problem or an issue autonomously, without the explicit help of your teacher		
**4.**	Learning, working in a group, in a collaborative environment		
**5.**	To be the main protagonist or responsible of your apprenticeship		
**6.**	Receiving a class, unconventional, in which the teacher acts only as a facilitator of the task		
**7.**	To use a learning methodology that can serve for different subjects or disciplines		
**8.**	Diagnosing what I need to know or learn in order to solve a problem or issue		
**9.**	To plan my learning process and the actions needed to solve an issue or problem		
**10.**	Integrate knowledge of different subjects or disciplines		
**11.**	Making decisions about what and how to learn		
