# Supplementary material for: Turning around Cycles: An Approach Based on Selected Problems/Cases to Stimulate Collaborative Learning about Krebs and His Four Metabolic Cycles
Source: J Chem Educ. 2022 May 25;99(6):2270–6. doi: 10.1021/acs.jchemed.1c01038 (PMC9202563; doi:10.1021/acs.jchemed.1c01038)
Supplement: Supplementary file 4 — ed1c01038_si_004.pdf [file ed1c01038_si_004.pdf]

PERCEPTION TEST ON KREBS AND HIS FOUR METABOLIC CYCLES PBL

Course: ________________ **Nickname:** _________________

#	Questions	TA	A	D	TD
**1**	I find this course useful and interesting.				
**2**	The "PBL" methodology used for the solving of the Krebs' cycle case is a novelty with respect to other ways of learning other topics of this same subject.				
**3**	The Krebs cycle case did not seem particularly complex to me and could be solved well mostly.				
**4**	I believe that the group's work dynamic has been efficient and satisfactory.				
**5**	I did not like working autonomously, without the teacher being directly responsible for my learning.				
**6**	The information provided in the Krebs' cycle case was relevant and sufficient to solve it.				
**7**	Sometimes we have been at a loss as to how to approach solving some part of the Krebs' cycle case.				
**8**	With this methodology (PBL) I have felt particularly involved.				
**9**	The length of the Krebs' cycle case did not seem adequate to me.				
**10**	I think we have been able to plan well the work of the group to solve the proposed case.				
**11**	With this methodology (PBL) I have not learned more than the traditional way of studying in this or other subjects.				
**12**	I have found the time required to solve the Krebs' cycle case to be adequate.				
**13**	This work methodology (PBL) has not required more work and preparation on my part than in others of the same or other subjects.				
**14**	In solving the Krebs' cycle case we have had the guidance of the professor whenever we have requested it.				
**15**	It seems to me that not all members of the group have worked with the same intensity and that some have taken advantage.				

(**TA**: Totally agree; **A**: Agree; **D**: Disagree; **TD**: Totally disagree)

(Please continue on the back...)

PERCEPTION TEST ON KREBS AND HIS FOUR METABOLIC CYCLES PBL

16. Overall, can you rate your degree of satisfaction with this activity?

☐- Not at all satisfied ☐- Not very satisfied ☐- Satisfied ☐- Very satisfied

17. After taking this course, what does Problem Based Learning mean to you?

____

____

____

____

____

____

____

____

____

____

18. Read and evaluate from 1 to 4 (1-minimum; 4-maximum) your level of experience and the importance you give to the following:

		Experience	Importance
**1**	To solve a problem or an issue autonomously, without the explicit help of your teacher		
**2**	Learning, working in a group, in a collaborative environment		
**3**	To be the main protagonist or responsible of your apprenticeship		
**4**	Receiving a class, unconventional, in which the teacher acts only as a facilitator of the task		
**5**	To use a learning methodology that can serve for different subjects or disciplines		
**6**	Diagnosing what I need to know or learn in order to solve a problem or issue		
**7**	To plan my learning process and the actions needed tc solve an issue or problem		
**8**	Integrate knowledge of different subjects or disciplines		
**9**	Making decisions about what and how to learn		
