# Supplementary material for: Turning around Cycles: An Approach Based on Selected Problems/Cases to Stimulate Collaborative Learning about Krebs and His Four Metabolic Cycles
Source: J Chem Educ. 2022 May 25;99(6):2270–6. doi: 10.1021/acs.jchemed.1c01038 (PMC9202563; doi:10.1021/acs.jchemed.1c01038)
Supplement: Supplementary file 5 — ed1c01038_si_005.pdf [file ed1c01038_si_005.pdf]

PBL (Problem or case-based learning)

Study of the Krebs cycles, their metabolism and regulation, and related diseases

1. Historical aspects of the scientific studies carried out by Sir Hans Krebs

1.4. You can also access the Nobel Lectures given by the two laureates. i) From Krebs' lecture, comment and explain the information provided by Table 1 and that provided by Figure 2. ii) In Krebs' lecture, an article by Severo Ochoa is mentioned. Identify it, look it up, put its full citation and add in an appendix the article by Ochoa downloaded from its source. Why does Krebs mention this article? What does this article contribute to the history of the Krebs cycle?

Group 3 (Biochemistry Grade) response: *i) Table 2 shows the results obtained by Stare and Baumann in a series of experiments confirming Szent-Györgyi's proposal that dicarboxylic acids (succinic, fumaric, malic and oxaloacetic acids) have a catalytic function. This is justified by the fact that with small amounts of these acids a considerable increase in respiration is achieved; moreover, this increase is proportional to the amount of oxygen needed to produce the oxidation of the incorporated acids. These scientists considered that dicarboxylic acids served as hydrogen transporters, but they could not explain the catalytic effect of these acids. Figure 2 shows the original scheme of the citric acid cycle, i.e., the first sequence of reactions proposed for this pathway. This carbohydrate oxidation pathway is characterized by the periodic formation of a series of di- and tri-carboxylic acids. The key finding for the outline of this pathway was the discovery that citrate could be produced from oxaloacetate and pyruvate or acetate, which allowed the cycle to be closed. The scheme describes the fate of the carbon atoms, as well as the steps in which hydrogen is removed and carbon dioxide is released. ii) Krebs mentions this article because thanks to the contributions of Ochoa and other researchers, the description of the citric acid cycle could be completed. Specifically in this article, Severo Ochoa talks about a previously unknown intermediate of the cycle, oxalosuccinate.*

This Nobel Lecture also cites other articles whose authors include Severo Ochoa. These were also of great importance in the history of the citric acid cycle, since they provided information about the role of acetyl-coenzyme A at the entry of the cycle, i.e., the pyruvate derivative that condensed with oxaloacetate to complete this pathway was identified.

1.6. Look up in the course content guides the context in which the different Krebs cycles are explained. With all the information handled, elaborate a brief essay on the contributions of the Krebs laboratory to the current knowledge of primary metabolism.

Group 10 (Biochemistry Grade) response: *Firstly, we will say that primary metabolism constitutes those biochemical processes directly involved in the survival, growth and reproduction of the organism, such as the citric acid cycle or glycolysis.*

Hans Krebs made a great contribution to the development and growth of knowledge in the metabolic field, specifically in the primary metabolic field, by describing several of the great cycles of primary metabolism, such as the urea cycle, the tricarboxylic acid (TCA) cycle and the glyoxylate cycle. These three cycles are connected by both fumarate and isocitrate. This intermediate is produced by argininosuccinate lyase activity and is also an intermediate of the Krebs cycle; on the other hand, from isocitrate, glyoxylate and succinate are generated by isocitrate lyase activity. This metabolite is capable of being incorporated into the TCA cycle for the formation of fumarate in the mitochondria.

2. On the structure and properties of some molecules involved in Krebs' cycles and on the toplogy of these cycles.

2.1. A very useful tool for the study of metabolic pathways is ***Wikipathways*** (URL: wikipathways.org). Search *Wikipathways* for the map for each of the three first Krebs' cycles, download them, insert them in your report and comment them.

Group 3 (Biology Grade) response: *Image 1. Krebs cycle in Populus trichocarpa. Anwesha B., Sacha B., Martina K. Kbres cycle (Populus trichocarpa) . <https://www.wikipathways.org/index.php/Pathway:WP2863>*

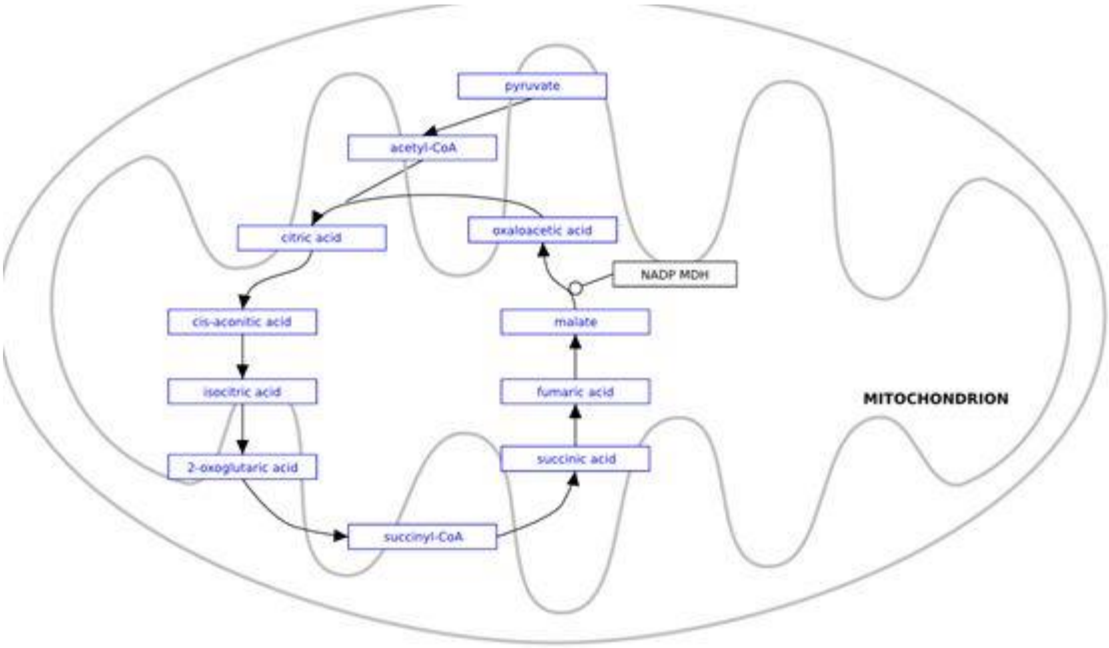

In this image we can see the Krebs cycle in Populus trichocarpa. There is a difference with the "classical" Krebs cycle and it is that in this plant the step from isocitrate to alpha-ketoglutarate does not occur, but in this step it forms 2-oxoglutarate from isocitrate (it should be noted that in this way it does produce NADH and C02 in the same way as in the "classical" route) and it will be this 2-oxoglutarate that gives rise to succinyl-CoA. The rest of the cycle as we can see would remain the same.

Image 2. Glyoxylate cycle in Mycobacterium tuberculosis. Andra Waagmeester, Kristina Hanspers, Martina Kutmon, Ferhart, et al. Glyoxylate cycle (Mycobacterium tuberculosis) . <https://www.wikipathways.org/index.php/Pathway:WP2566>

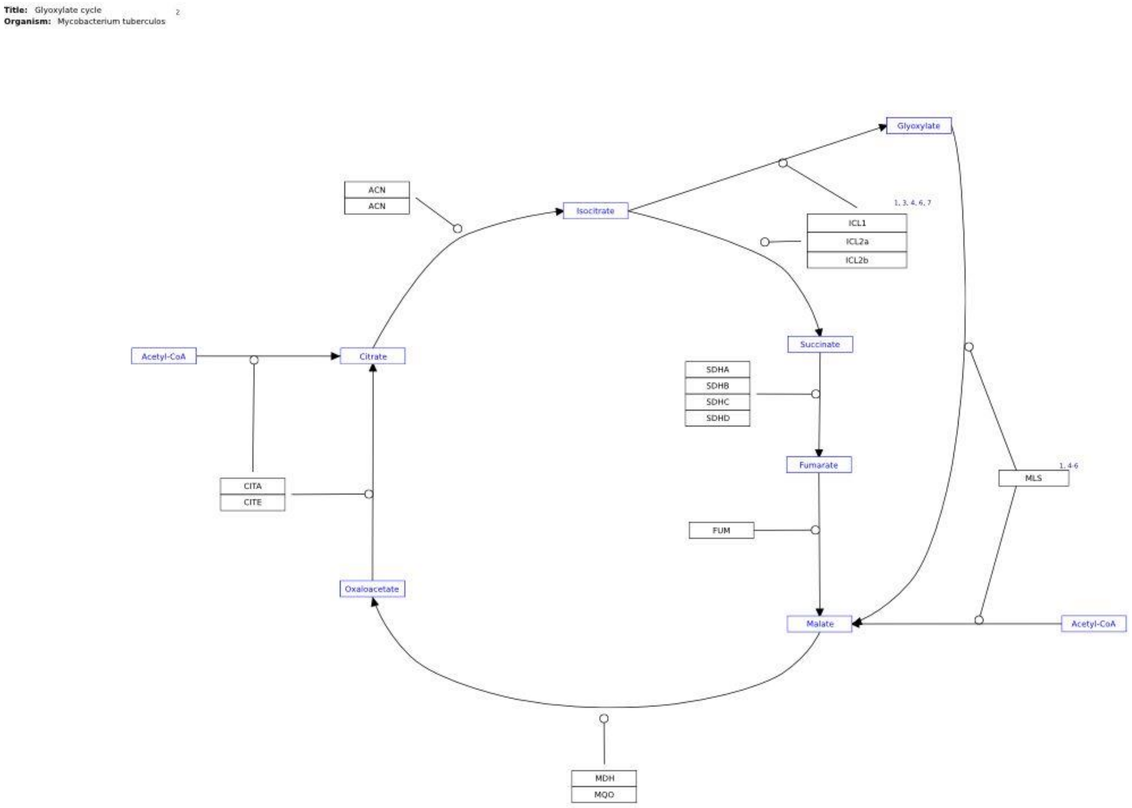

As we can see in the image, isocitrate would give rise to glyoxylate which in turn would transform into malate instead of first passing through the succinate and fumarate forms, and after reaching malate the cycle would proceed like the Krebs cycle until returning to isocitrate.

Image 3. Martina Kutmon. Urea cycle and metabolism of amino groups (Bos taurus). < https://www.wikipathways.org/index.php/Pathway:WP3245>

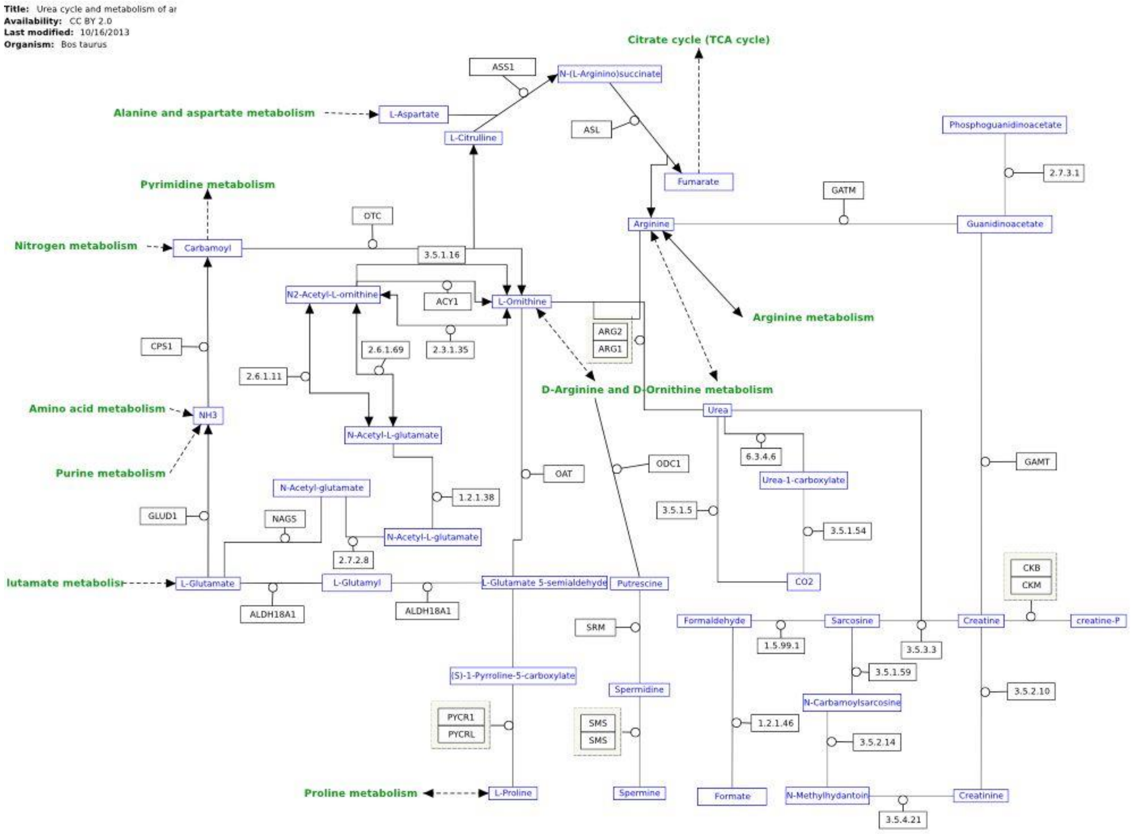

In the image we see the urea cycle or also known as the ornithine cycle, since it is from this compound where it starts. In this particular scheme, we can see a number of enzymes and pathways that interact with this cycle. Ornithine gives rise to citrulline that when receiving the incorporation of an aspartate group originates arginine succinate that will decompose giving rise on the one hand to fumarate that will be used in the Krebs cycle and on the other hand also gives rise to arginine that decomposes in turn to give rise to urea and again to ornithine closing the cycle.

2.5. ***PDB*** is a freely accessible database that provides information on the three-dimensional structure of proteins. You can access the European *PDB* portal at the URL: ebi.ac.uk/pdbe. Search in *PDB* some image of the crystalline structures of citrate synthase, malate synthase and arginine succuniate lyase. Include in your report the downloaded images, their respective PDB references and a brief comment to each one.

Group 4 (Biology Grade) response: *Crystal structure of citrate synthase: enzyme present in almost all cells. In eukaryotes it is located in the mitochondrial matrix. Catalyzes the first reaction of the Krebs cycle, where acetate from acetyl-CoA is condensed together with oxaloacetate to produce citrate and CoA-SH. https://www.ebi.ac.uk/pdbe/entry/pdb/2c6x.*

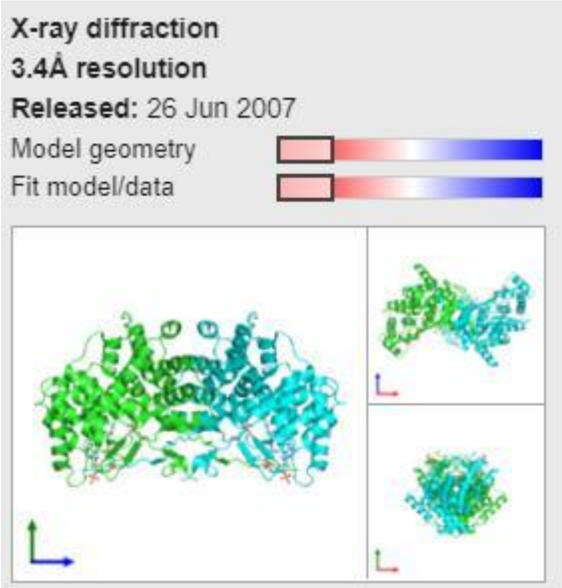

Crystal structure of malate synthase: it is an enzyme which transfers the acetyl-CoA transfers an acetyl group to glyoxylate producing malate. https://www.ebi.ac.uk/pdbe/entry/pdb/5cah

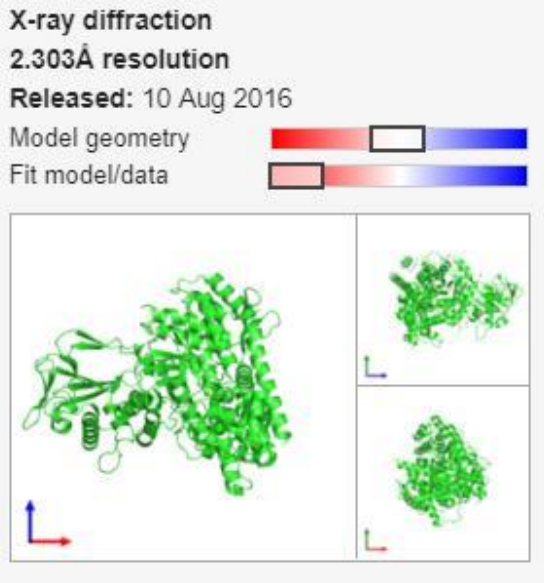

Crystal structure of argininosuccinate lyase: an enzyme that catalyzes the synthesis of argininosuccinate from citrulline and aspartate. It is responsible for the third step of the urea cycle and some reactions of the citrulline-NO cycle. https://www.ebi.ac.uk/pdbe/entry/pdb/1k97

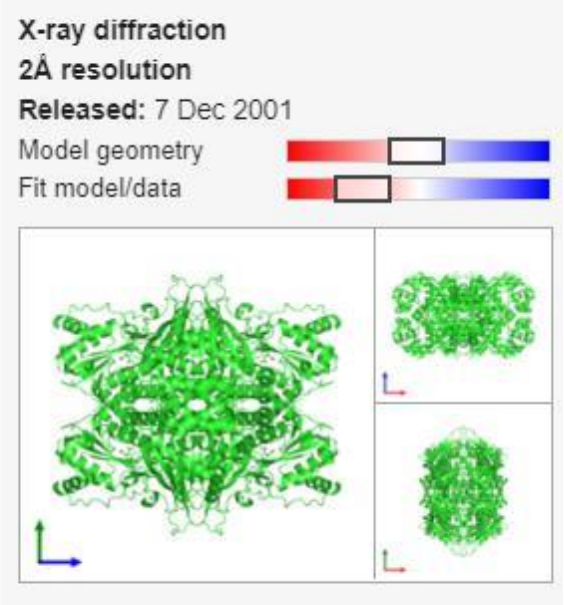

2.6. A very useful tool to understand in more detail the structure and properties of chemical compounds is ***PubChem*** (URL: pubchem.ncbi.nlm.nih.gov). Search in *PubChem* for the information it contains about the structure and properties of acetyl-CoA, glyoxylate and urea, and extract the most relevant information to include in your report.

Group 11 (Biochemistry Grade) response: *Acetyl-CoA: Acetyl-CoA is involved in the synthesis of fatty acids and sterols, in the oxidation of fatty acids, in the metabolism of innumerable amino acids and in the metabolism of carbohydrates. Its acetyl group is used in the Krebs cycle for further oxidation to obtain energy. It is also a biological agent of acetylation. It is the precursor of HMG-CoA, which forms cholesterol and ketone with acetyl-CoA and acetoacetyl-CoA by HMG-CoA synthase.*

In relation to its structure this coenzyme consists of a cysteamine which is linked to pantothenic acid by an amide bond and a 3´-phosphorylated adenosine phosphate. The cysteamine has a thiol in its structure that is attached to the acetyl group. The thioester bond is a highly energetic bond (-31.5Kj/mol, exergonic). Coenzyme A is formed by its acetylation to acetyl-CoA by the degradation of carbohydrates in glycolysis and by degradation of fatty acids in β-oxidation. The acetyl group in the Krebs cycle is oxidized to give carbon dioxide and H2O , giving off energy in the form of 11 ATP and 1 GTP. Konrad Bloch and Feodor Lynen won the 1964 Nobel Prize in Physiology and Medicine for "their discoveries linking acetyl-CoA and fatty acid metabolism". Fritz Lipmann won the Nobel Prize in 1953 for "his discovery of the cofactor coenzyme A". It belongs to the organic group of hydantoin. Its molecular formula is C23H 38N 7O 17P 3S.

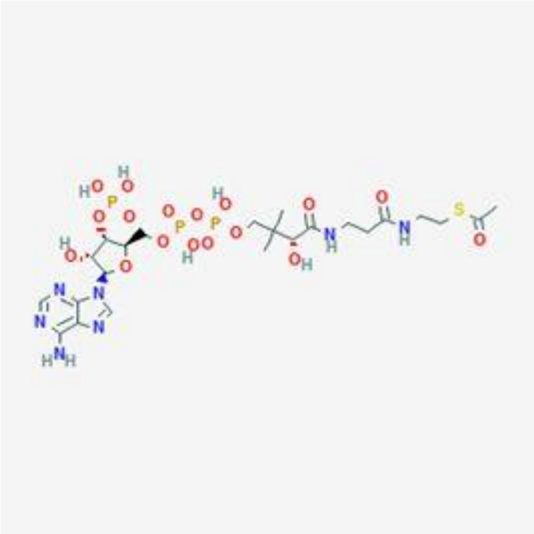

Glyoxylate: Glyoxylic acid is an organic compound that can be classified as an aldehyde or as a carboxylic acid. It is an intermediate of the glyoxylate cycle that converts fatty acids into carbohydrates in certain organisms (bacteria, protozoa and plants). This compound is formed by two pathways: by the catabolism of hydroxyproline in mitochondria and by the oxidation of glycolate in peroxisomes. It can act as a nephrotoxin and metabotoxin in some specific circumstances. In relation to its structure, it is a 2-oxo monocarboxylic acid, i.e. an acetic acid with an oxo group on the alpha carbon. Its molecular formula is C2H2O3.

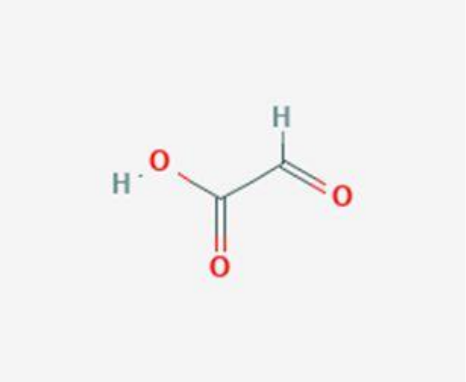

Urea: Urea is a nitrogenous compound formed by the liver from ammonia due to the deamination of amino acids. It is the end product of protein catabolism. This urea is formed in the liver by two ammonium molecules with one CO2 molecule in the urea cycle.

In relation to its structure it has two amino groups attached to a carbonyl group which has osmotic diuretic activity. Urea raises plasma osmolality, which accelerates the flow of water in the tissues. It is used as a fertilizer and industrially. Its molecular formula is CH4N2O.

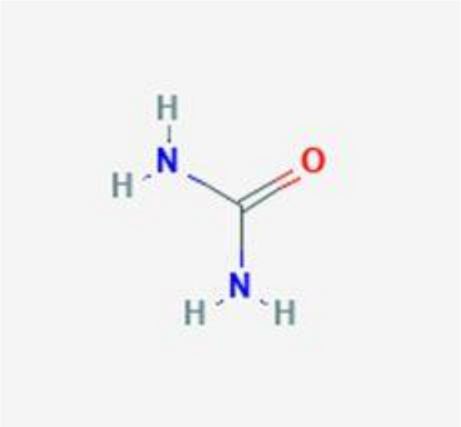

2.10. ***The Krebs bicycle*** was an initiative encouraged by the five students that make up the "Krebs bicycle" team (Hugo Pineda, Lola Nevado, Sara Bernárdez, María Jesús Pacheco, Juan Jesús Criado and Florencio Palomas) and that achieved the active participation of more than half of the students enrolled in the 2012-13 academic year in the Metabolic Biochemistry course of the former Bachelor's Degree in Biology. In the words of the promoters of this initiative: "Who hasn't thought at some point: 'What a boring class'? Couldn't there be a way to learn this in a more enjoyable and fun way? Although we are sometimes led to believe that there isn't, there is". Krebs' bicycle is a good example that there are positive answers to this last question. An answer, moreover, that in this case has been found, created, developed and carried out by the students themselves in the format of a radio program. The first monographic issue of ***Encounters in Biology*** of the year 2014 collects this happy student initiative, including the transcription of all the contents of the radio magazine that had its "world premiere" at the Botanical Garden of the University of Malaga on Saturday, June 1, 2013. Download this monographic issue of Encounters in Biology from the Virtual Campus of the subject, read its contents and use the links if you want to listen to them. Finally, write an essay commenting on the highlights of the experience.

Group 2 (Biochemistry Grade) response: "*The Krebs bicycle" was an initiative of some students of the biology degree, which arose with the aim of making metabolism easier and more enjoyable to learn. Although the content consulted is in writing, it was actually a radio recording. The work is inspired by the Krebs cycle, which consists of two interrelated cycles: the urea cycle and the citric acid cycle. Both were described by Hans Krebs.*

The podcast begins with a kind of newscast, in which, as a news item, some relevant current news is presented. One of them, for example, is the creation of a "Google map of metabolism". This is a complete map of metabolism in which information from the study of 65 types of human cells and about 7000 reactions has been included. Also, the discovery that some ethnic groups assimilate less nicotine from tobacco, which explains why they are less likely to suffer from lung cancer. In addition to these, several other topical scientific news items are discussed in this section.

A tribute is paid to a French biologist, Françoise Jacobs, who was awarded the Nobel Prize in physiology and medicine in 1965 along with other scientists, for his discovery of genetic control in the synthesis of enzymes and viruses.

After this, there is a "pause" in the podcast, in which a song is played, called the mitochondrion, by the group The Plastoquinones and The free Energy Band.

Subsequently, a section is opened as an interview on rare diseases, in which some of these, which are closely related to metabolism, are going to be discussed.

First we talk about trimethylaminuria or fishy odor syndrome, which is a metabolic disorder

metabolic disorder characterized by a body odor similar to rotten fish. This only manifests itself once the child is weaned and begins to eat foods containing trimethylamine precursors. In these people the gene for monooxygenase 3 is mutated, there is a defect in the metabolism of trimethylamine, causing the excretion of this amine in sweat and other secretions. There is no cure.

Another is congenital insensitivity to pain, a disease that is a hereditary sensory neuropathy, which causes the patient to be insensitive to stimuli and self-mutilation. In addition, there is a selective loss of small myelin fibers.

Fumaric aciduria is a deficiency of fumarase, which catalyzes the passage of fumarate to malate in the Krebs cycle. This causes an excessive accumulation of fumarate, resulting in low muscle tone and excess amniotic fluid.

Finally, cystinosis, whose symptoms are growth retardation, hypothyroidism and enlargement of the liver and spleen, is caused by the accumulation of cystine in the lysosomes in some tissues, due to a defect in its transport, a defect caused by a mutation in the gene encoding the enzyme cystinosin (lysosomal membrane protein).

In addition, there are two syndromes: the first, Smith-Lemli-Opitz syndrome, is a congenital anomaly in cholesterol synthesis. Symptoms include multiple congenital anomalies and behavioral problems.

Zellweger syndrome is a perixosomal metabolic syndrome, most commonly caused by a genetic defect. When the function of the peroxisome is altered, an accumulation of long-chain fatty acids occurs. People with this syndrome present facial malformations, and

visual, auditory and sensory problems, among others.

After the section on rare metabolic diseases, the metabolic carousel section begins. In this section, certain metabolic pathways are treated as if they were sports.

The next section is called gastronomy and nutrition, which is included in this podcast because it has a tremendously relevant role in metabolism. Here the topic is discussed in the form of an interview, and we talk about the controversial topic of the Duncan diet and molecular gastronomy.

Molecular gastronomy is the application of science to culinary practice, taking advantage of the physicochemical properties of food to innovate and create new combinations. Spherification, for example, is a technique in which spheres that simulate roe are created and filled. The spheres are created thanks to a mixture of alginate and juice. This is pipetted into a CaCl2 solution. It is the calcium that causes the gelling. Ferrán Adriá is one of the pioneering chefs of this culinary technique.

We now turn to the Duncan diet, which is a diet based on the intake of large amounts of protein. It has several phases: attack phase, cruising phase, consolidation phase and last phase. If we analyze the cost at the metabolic and physiological level of our body, this diet is not so beneficial. This is due to the rebound effect, the formation of ketone bodies due to excessive protein intake and deficiency in essential vitamins.

Now, the podcast continues with the metabolic carousel. It continues to discuss carbon assimilation in the Calvin cycle.

The next section is biochemistry on the go, a section that brings biochemistry closer to everyday life. First, taurine, which is a common component in energy drinks, is discussed. Taurine is an organic acid involved in the formation of bile, which occurs naturally in some tissues. It is a free amino acid, abundant in tissues with high electrical activity, and whose blood levels in

activity, and whose blood levels decrease with age. There are certain diseases that may be related to deficiencies or increased requirements of this amino acid. Taurine has a great therapeutic potential, for example, by reversing the stiffening effect of tobacco consumption on blood vessels. Thus, it is seen that the restriction in the consumption of energy drinks should be

due to the high amount of sugars and caffeine they contain. Taurine on the other hand is necessary, and in excess can be eliminated in the urine, so it really has no negative effects. However, energy drinks should not be mixed with alcohol, as mixing depressants with stimulants can cause abnormal heart rhythms (which would have consequences in the future) and sudden death.

The next section is Metabolic Maximum, which is a fictitious interview with Linus Pauling, a bit satirical. Then a new song by The Plastoquinones & The Free Energy Band, in this case called replication.

The animal curiosity section deals with some of the curiosities of the metabolism of certain animals depending on the environment in which they find themselves. First they talk about tardigrades, which are capable of losing up to 99% of the water in their bodies, in order to adapt to the cycles of humidity and drought. They achieve this by reaching the state of inanimate suspension or anhydrobiosis. In this state they reduce their metabolism by 99.99%. Sometimes they have remained in this state even for a hundred years. They also do cryptobiosis, which is the reduction of their metabolism in order to survive extreme environmental conditions.

Another example of an animal involved is the Rana silvatica, which is able to freeze itself several times and for a long time. When it thaws, the heart thaws first, and then the other structures.

structures.There is also talk of the sea slug, an animal that is capable of photosynthesis. It does so by "stealing" chloroplasts from the algae it consumes, and also genes responsible for chlorophyll production. Also a multicellular animal that is able to survive in the total absence of oxygen, in fact, there are three animal species, which are based on carbon metabolism.

Another section is the metabolic do you know/don't know, a quiz in which a version of the program of the same name is created, but with questions on metabolism.

In the section called "World of Margaritas", an interview was conducted with a professor of ecology at the University of Malaga. This professor talks about geometabolism or global metabolism, he relates the metabolism of a global ecosystem that is the Earth, which he calls "Gaia". In this way, global metabolism is not the metabolism of each one of the parts but more than that, as other emergent properties of global metabolism emerge. He then discusses the metabolism of aquatic organisms and explains the difference between autotrophic and heterotrophic organisms, according to the relationship between carbon production and respiration. In addition, he presents us that climate change is breaking the balance between carbon production and respiration, thus creating a negative feedback for the greenhouse effect.

In the section called "Metabolism of Andalusia" we talk about some research groups at the University of Malaga and what they do.

In the section called "Connected" they will talk about the Krebs cycle, as it is a so-called metabolic hub. It is the confluence of different routes of the metabolic network. In addition, it is an amphibolic pathway, which acts in both catabolic and anabolic processes, many catabolic processes generate Krebs cycle intermediates.Tehy also talk of ketogenic amino acids, which give rise to acetyl CoA or in part, or glycogenic amino acids, those that form oxaloacetate or those that form pyruvate. Or other intermediates upstream of oxaloacetate. The Krebs cycle is also connected to the urea cycle, and this is known as the Krebs cycle. In addition, the Krebs cycle is an anaplerotic pathway, i.e., reactions that have constant cycle intermediates. They also conduct a fictitious interview with Succinyl-CoA, and its metabolization process. He tells us about the chemical changes that take place until Pyruvate is formed. In addition, he tells us about other intermediates of the Krebs cycle metabolon.

In the section called "LSD" the discoverer of the drug LSD A.Hoffman in 1938, tells us about his first experiences and symptoms and how it was his discovery. After this, years later in 1963 LSD "hit the street" and was called elixir. There was a time when it could be bought in pharmacies, but

after some negative symptoms it was outlawed by the U.S. authorities, and from this a black market arose, with the adulteration of the drug.

3. On the four metabolic Krebs cycles, their regulation and their metabolic integration.

3.3. The urea cycle is a compartmentalized pathway between cytoplasm and mitochondria. This implies the necessary involvement of inner mitochondrial membrane transporter systems in the process. However, the maps of the cycle depicted in biochemistry textbooks do not emphasize this fact. i) Find which transporter systems are integral to the urea cycle. Download Figure 4 from the review by **Bröet and Palacín. Biochem J 436: 193-211, 2011** and comment on it, highlighting the central role of the ORC transporter system and the collateral role of AGC, OGC and CIC in the urea cycle. ii) Search PDB and GeneCards (https://www.genecards.org) for information on ORC structure and function.

Group 2 (Biology Grade) response: *i)*

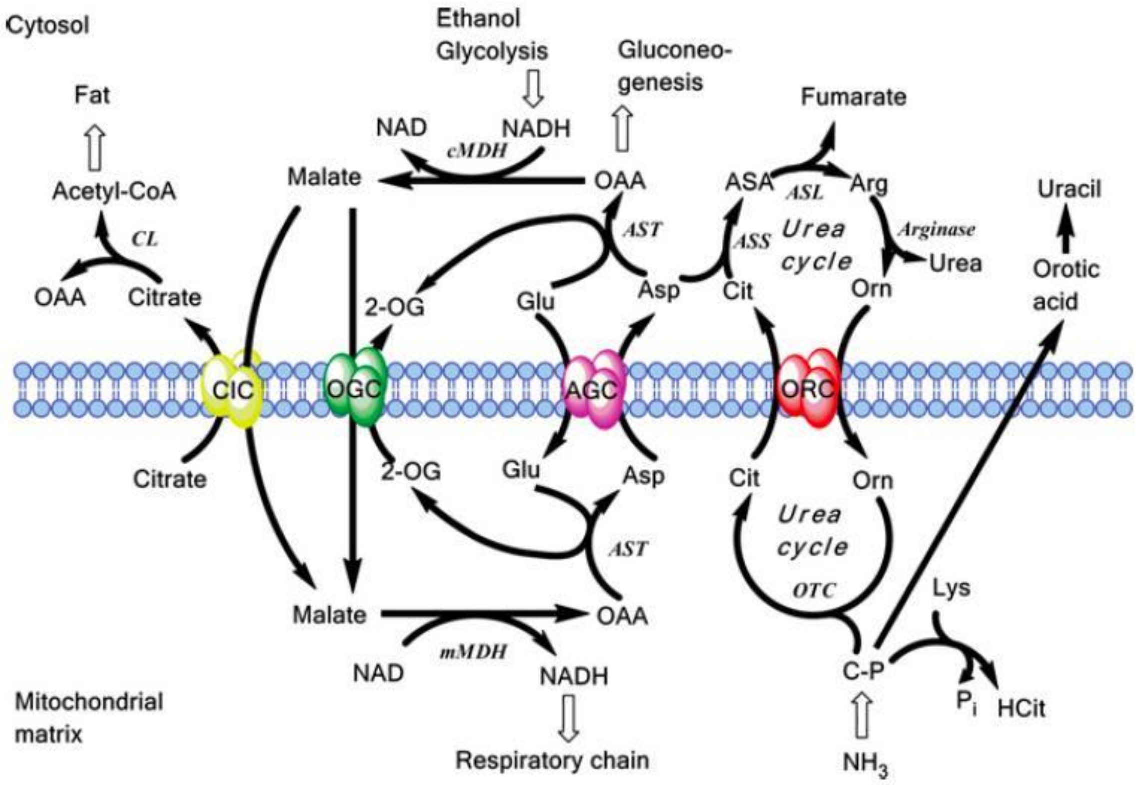

This scheme represents the different transporter systems that form an integral part of the urea cycle.

First of all, the central role of the ORC transporter system must be emphasized, which is responsible for catalyzing the transport of ornithine (from the outside to the mitochondrial matrix) in exchange with citrulline (from the mitochondrial matrix to the outside) across the inner mitochondrial membrane; it is an inner mitochondrial membrane antiport directly involved in the urea cycle.

In the inner mitochondrial membrane we find 2 variants of this antiport: ORC1 (SLC25A15) and ORC2 (SLC25A2); both exchange the L-stereoisomers of ornithine, lysine, arginine and citrulline with a 1: 1 stoichiometry (although ORC2 has lower specificity and accepts L- and D-histidine, L-homoarginine and D-stereoisomers of ornithine, lysine and arginine as substrates).

ORC1 is thought to be the main mediator of this exchange because of its higher expression levels in liver and other tissues (such as lungs, pancreas and testis).

The collateral role of AGCs, OGCs and CICs indirectly involved in the urea cycle is highlighted below.

AGC1 (SLC25A12) is the transporter responsible for catalyzing the transport of aspartate (from the mitochondrial matrix to the outside) in exchange (antiport) with glutamate (from the outside to the mitochondrial matrix). This antiport is thought to be necessary for mitochondrial oxidation of NADH in the brain.

OGC (SLC25A11) is the oxoglutarate transporter (from the mitochondrial matrix to the cytosol), and is also responsible for importing malate into the mitochondrial matrix.

CIC (SLC25A1) is the citrate transporter from the mitochondrial matrix to the cytosol. It also transports malate from the cytosol to the mitochondrial matrix. Citrate can be utilized in other metabolic pathways, both to enter the lipid pathway and to give oxaloacetate.

ii) ORC refers to the mitochondrial ornithine transporters (ORNTs), which are two proteins in the inner mitochondrial membrane of animals that catalyze the transport of ornithine in exchange with citrulline across the membrane. This reaction is part of the urea cycle. Both proteins contain 301 amino acids, and are 88% identical.

3.8. In facultative anaerobic organisms the "complete" Krebs cycle functions under aerobic conditions, whereas in anaerobiosis the cycle becomes a branched biosynthetic pathway. On page 18 of the book "***Metabolic Reprogramming as a Target for Cancer and Other Diseases***", downloadable from the institutional repository of the UMA (https://riuma.uma.es/xmlui/) you can find a figure that illustrates the reprogramming of energy metabolism in yeast. i) Download the figure, include it in the report and comment on it. ii) Write down the complete citation of the article by **Mayor Zaragoza**'s group that demonstrated this in the first place. Comment on the contents of that article.

Group 9 (Biochemistry Grade) response: *i) In this figure we can see the two variants of the Krebs cycle that can occur depending on the presence of oxygen. On the left we have the cycle as we know it, while on the right we have the result that occurs in anaerobic conditions. As can be seen, in this case the cycle is broken by the absence of the 2-oxoglutarate dehydrogenase complex. The result is a branched biosynthetic pathway capable of synthesizing compounds with 4 carbon atoms (when we go from oxaloacetate to succinate) and also compounds with 5 carbon atoms (if we go from oxaloacetate to α-ketoglutarate).*

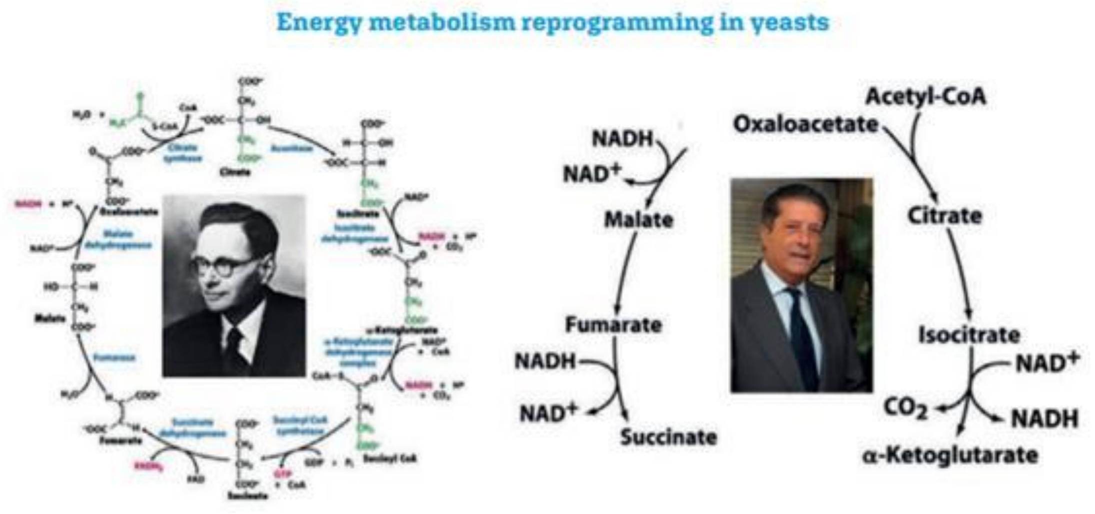

ii) Machado, A., Núñez de Castro, I. & Mayor, F. Isocitrate dehydrogenases and oxoglutarate dehydrogenase activities of baker's yeast grown in a variety of hypoxic conditions. Mol. Cell. Biochem. ***6****, 93–100 (1975).*

The activities of the enzymes isocitrate dehydrogenase (NAD), isocitrate dehydrogenase (NADP) and oxoglutarate dehydrogenase of Saccharomyces cerevisiae under a variety of aerobic and hypoxic conditions are studied. The effects of high glucose concentrations, addition of protein synthesis inhibitors in mitochondria, respiratory inhibition by azide, and mutants with impaired respiration are also studied.

All hypoxic conditions lead to a marked decrease in oxoglutarate dehydrogenase and significant decreases in both isocitrate dehydrogenases. According to its kinetic properties, NAD-isocitrate dehydrogenase will not be functional in hypoxia "in vivo". Because of this and other related facts, it is concluded that hypoxia under yeast conditions generally leads to an unfolding of the tricarboxylic acid cycle and that glutamate synthesis under these conditions takes place through the coupling of NADP-linked isocitrate and glutamate dehydrogenase.

3.10 Duncan Bootland wrote an essay for a biochemistry course by Professor D.A. Bender in story format. Professor Bender liked it so much that he sent it for publication with his student's authorship in the journal ***Biochemical Education (26. 14-15, 1998***). ii) Dare to write a story with the title "We are all malate dehydrogenase!" (mentioned in 3.7).

Group 2 (Biology Grade) response: *My family and I work in a factory called Krebs cycle in which 7 other families work, which have very important functions to maintain the city we belong to, The Cell.*

I belong to the citrate synthase family. Our main job is to turn on the power plant, thanks to acetyl-CoA that the city next door, Citoplasma, gives us every morning. Our power plant has a large pipe that collects water from the river that surrounds the factory, thanks to the water and the acetyl-CoA that our neighbors give us, we are able to produce citrate, a very important molecule for the factory, and without which it could not produce enough energy to maintain The Cell, but the factory produces waste as coenzyme A that is dumped into the river every day.

My best friend is family of the aconitase, they work in another part of the factory, they are in charge of dehydrating the citrate that we send them, to produce cis-aconitrate, and with this they produce clean water that they use again to hydrate the cis-aconitrate and produce isocitrate.

The isocitrate dehydrogenase, use the NAD+ that also sends us cytosol, to produce an oxidative decarboxylation in the isocitrate that makes my best friend, the aconitase, and finally produce alpha-ketoglutarate, but always produce many CO2 emissions that is polluting for The Cell, also generate NADH+H+ that will use later those of the factory of the oxidative phosphorylation to produce ATP that need all the inhabitants of The Cell.

I hardly know them, but I know that the alpha-ketoglutarate dehydrogenase, work intensively every day to oxidatively decarboxylate the alpha-ketoglutarate made by the isocitrate dehydrogenase, thanks as before to the NAD+ of the cytosol, to give succinyl-CoA, do you remember the coenzyme A waste that I told you we were pouring into the river? Well, they use them again, and in this plant we recycle everything we can, but again, they are in charge of producing more CO2, but also NADH+H+ for the oxidative phosphorylation factory.

Next to the alpha-ketoglutarates work the succinyl-CoA synthetases, which take the succinyl-CoA given to them by the previous ones, they break the union of CoA with succinyl and leave each one on its own side as CoA and succinate, besides they are tremendously important because they generate energy in the form of ATP and GTP for The Cell.

My boyfriend, for example, works together with his family, the succinate dehydrogenase, in dehydrogenating the succinate from before to obtain fumarate, they do this because we have some very important reservoirs of reducing molecules that help them, the FADs.

The problem comes now, and is that we have been in crisis in the factory for a few weeks, and the bosses want to fire the malate dehydrogenase, but we have told them that they are very important for us, especially for my family, they are the ones who provide us with the oxaloacetate that we use every morning to run the factory, if it were not for them doing the last shift of the day, all our effort would be meaningless.

So all the factory workers have gone on strike under the slogan "We are all malate dehydrogenase!". We want to make the owner understand, that without them The Cell would not work and that because of that illnesses would occur that would affect our whole world, The Human Body.

3.21. Prepare an essay detailing the integration of fermentative and oxidative metabolism in a growing tumor.

Group 1 (Biochemistry Grade) response: *In tumors, the rate of glycolysis and the capacity to uptake glucose is higher than in non-tumor cells. In such tumor cells, the so-called Warburg effect or aerobic glycolysis occurs, whereby the cells metabolize glucose to lactate even in the presence of oxygen. Why do tumor cells take the fermentative pathway as opposed to the energetically more efficient oxidative phosphorylation? First, lactic fermentation acidifies the tumor environment via lactic acid, facilitating tumor invasion and inhibiting the tumor-attacking immune system. Second, increased glycolytic uptake and formation of glucose 6-phosphate provides substrates for the pentose phosphate pathway, which together with glycolysis, provides reducing power and energy for the biosynthesis of macromolecules such as nucleotides, necessary for tumor growth.*

Cancer cells, growing faster than the blood vessels that feed them, begin to experience hypoxia, i.e. oxygen deficiency. The Warburg effect reduces the oxygen dependence of cancer cells. Hypoxia increases the expression of the HIF-1 gene, which increases aerobic glycolysis and allows increased tumor vascularization.

The biochemical alterations that induce the Warburg effect, i.e. the shift in metabolism from cellular respiration to aerobic glycolysis, remain unclear, but all point to mutations in genes expressing glycolytic enzymes, such as hexokinase and pyruvate kinase.

4. Diseases linked to a bad functioning of the Krebs metabolic cycles. Biochemical substantiation of clinical cases.

4.3. ***OMIM*** (Online Mendelian Inheritance in Man) is an extensive online catalogue (URL: omim.org) of human genes and genetically based diseases, including inborn error of metabolism. On the other hand, the most important database portal for rare diseases is ***Orphanet*** (URL: orpha.net). a) Search for the OMIM and ORPHA codes corresponding to each of the diseases mentioned in 4.1. b) Download a summary sheet with the most important information associated with these diseases in OMIM and Orphanet. c) Summarize the differential features of DOOR syndrome.

Group 1 (Biology Grade) response: *a-b)*

Mitochondrial encephalopathy. ORPHA: 550 OMIM: 540000

MELAS

Suggest an update

Disease definition

MELAS (Mitochondrial myopathy, encephalopathy, lactic acidosis, and stroke) syndrome is a rare progressive multisystemic disorder characterized by encephalomyopathy, lactic acidosis, and stroke-like episodes. Other features include endocrinopathy, heart disease, diabetes, hearing loss, and neurological and psychiatric manifestations.

ORPHA:550

Synonym(s):

Mitochondrial encephalomyopathy, lactic acidosis and stroke-like episodes

Mitochondrial myopathy, encephalopathy, lactic acidosis and stroke-like episodes

Prevalence: **1-9 / 1 000 000**

Inheritance: **Mitochondrial inheritance or Not applicable**

Age of onset: **Adolescent, Adult, Childhood**

ICD-10: **G71.3**

OMIM: 540000

UMLS: **C0162671**

MeSH: **D017241**

GARD: 7009

MedDRA: **10053872**

Ethylmalonic encephalopahyt. ORPHA: 51188 OMIM: 602473

Disease definition

Ethylmalonic acid encephalopathy (EE) is defined by elevated excretion of ethylmalonic acid (EMA) with recurrent petechiae, orthostatic acrocyanosis and chronic diarrhoea associated with neurodevelopmental delay, psychomotor regression and hypotonia with brain magnetic resonance imaging (MRI) abnormalities.

ORPHA:51188

Synonym(s): -

Prevalence: **<1 / 1 000 000**

Inheritance: **Autosomal recessive**

Age of onset: **Infancy, Neonatal**

ICD-10: **G31.8**

OMIM: 602473

UMLS: **C1865349**

MeSH: **C535737**

GARD: 2198

MedDRA: -

Fumaric aciduria. ORPHA: 24 OMIM: 606812

Disease definition

Fumaric aciduria (FA), an autosomal recessive metabolic disorder, is most often characterized by early onset but non-specific clinical signs: hypotonia, severe psychomotor impairment, convulsions, respiratory distress, feeding difficulties and frequent cerebral malformations, along with a distinctive facies. Some patients present with only moderate intellectual impairment.

ORPHA:24

Synonym(s): Fumarase deficiency

Prevalence: <1 / 1 000 000

Inheritance: Autosomal recessive

Age of onset: Infancy, Neonatal

ICD-10: E88.8

OMIM: 606812

UMLS: C0342770 C2936826

MeSH: C538191

GARD: 6476

MedDRA: -

DOOR síndrome. ORPHA: 79500 OMIM: 220500

Disease definition

DOORS syndrome (also known as DOOR syndrome) is a multiple congenital anomalies-intellectual disability syndrome characterized by sensorineural hearing loss (deafness), onychodystrophy, osteodystrophy, mild to profound intellectual disability, and seizures.

ORPHA:79500

Synonym(s):

Autosomal recessive deafness-onychodystrophy syndrome

DOOR syndrome

Deafness-onychodystrophy-osteodystrophy-intellectual disability syndrome

Deafness-onychodystrophy-osteodystrophy-intellectual disability-seizures syndrome

Deafness-onychoosteodystrophy-intellectual disability syndrome

Prevalence: <1 / 1 000 000

Inheritance: Autosomal recessive

Age of onset: Neonatal, Antenatal

ICD-10: Q87.8

OMIM: 220500

UMLS: C0795927

MeSH: -

GARD: 1685

MedDRA: -

c) DOOR syndrome is a disease that usually affects newborns, it generates sensorineural deafness due to the It causes neurosensory deafness due to the brain stem, lack of cutaneous appendages such as nails, shortening or loss of the normal number of of the normal number of phalanges of hands and feet, cases of blindness, slow body development, permanent convulsions that increase progressively with the years, diminution of the corporal motor activity, it is not progressive. motor activity, it is not progressive and they present an increase of the width of the nasal bridge. nasal bridge. There are also other characteristics that are rarer to be found but which may occur if the disease has affected more severely, however, it is not progressive. There

is no specific treatment for it but the ways to combat it are treated separately for each aspect that it affects. each aspect it affects.

4.5. i) Use OMIM, Orphanet or PhenUMA to find out if there is any disease associated with a defect in the functioning of any of the enzymes involved in the uric acid cycle. Comment your results.

Group 4 (Biochemistry Grade) response:

Pathologies associated with the uric acid cycle:

- <u>Kelley-Seegmiller syndrome.</u>

The disease is caused by a partial deficiency of HPRT due to mutations in the HPRT1 gene (Xq26). Inheritance is X-linked recessive. UAO may be due to impaired recycling of purine bases with increased synthesis of purine nucleotides leading to hyperuricemia which increases the risk of precipitation of UA crystals in tissues to form tophi, in joints leading to inflammatory processes and gouty arthritis, and renal UA excretion causing urolithiasis. Virtually complete deficiency of residual HPRT activity is associated with Lesch-Nyhan syndrome (LNS), whereas partial deficiency (at least 8%) is associated with Kelley-Seegmiller syndrome. LNS is characterized by abnormal metabolic and neurologic manifestations. In contrast, Kelley-Seegmiller syndrome is usually associated only with clinical manifestations of excessive purine production. Kidney stones, uric acid nephropathy, and renal obstruction are often the presenting symptoms of Kelley-Seegmiller syndrome, but rarely of LNS. After puberty, hyperuricemia in Kelley-Seegmiller syndrome may cause gout.

The exact prevalence is unknown, but is probably underestimated due to misdiagnosis. KSS may account for about 15% of patients with HPRT deficiency.

The age of onset is usually in childhood, but can also be in adulthood (up to 30 years). Males are usually affected and heterozygous females are carriers (usually asymptomatic). Patients are normal at birth. The first manifestation is the presence of orange crystals in diapers. Urolithiasis, uric acid nephropathy, urinary tract infections and renal obstruction are often the presenting symptoms. Gout may appear after puberty with acute arthritis or tophi. In contrast to Lesch-Nyhan syndrome (LNS; see this term), dystonia may be mild or even absent. Patients have normal intelligence associated with varying degrees of attention deficit. Compulsive self-aggressive behavior is absent. With appropriate treatment, renal function remains stable and patients have a normal life expectancy.

- <u>Lesch-Nyhan syndrome</u>

Virtually complete deficiency of residual HPRT activity (less than 1.5%) is associated with Lesch-Nyhan syndrome, whereas partial deficiency (at least 8%) is associated with Kelley-Seegmiller syndrome. LNS is characterized by abnormal metabolic and neurologic manifestations. In contrast, the Kelley-Seegmiller syndrome LNS, with uric acid overproduction and neurologic disability ranging from minor clumsiness to debilitating pyramidal and extrapyramidal motor dysfunction.

There is variable disease severity in patients with Lesch-Nyhan syndrome, with an inverse relationship between HPRT1 enzyme activity measured in intact cells and clinical severity. Patients with classic Lesch-Nyhan disease, the most severe and frequent form, have the lowest HPRT enzyme activity (less than 1.5% of normal) in intact cultured fibroblasts. Patients with partial HPRT deficiency, designated as Lesch-Nyhan variants, have HPRT1 enzyme activity ranging from 1.5 to 8.0%. Individuals with an intermediate variant known as the "neurological variant" are neurologically indistinguishable from patients with Lesch-Nyhan disease, but have no self-injurious behaviors and normal or near-normal intelligence. Less affected patients with the variant

have residual HPRT1 enzyme activity greater than 8%. Inheritance is X-linked recessive and genetic counseling is essential.

The estimated prevalence at birth is between 1 / 380,000 and 1 / 235,000 live births. Males are usually affected and heterozygous females are carriers (usually asymptomatic). Patients are normal at birth. Psychomotor delay becomes evident within 3 to 6 months, with delayed head support and sitting, hypotonia and athetoid movements. Sandy urine in diapers or crystalluria with urinary tract obstruction are common forms of presentation. Patients have severe action dystonia with basal hypotonia that may lead to an inability to stand and walk, and involuntary movements (choreoathetosis and ballismus) associated with voluntary movements increase with stress, but are not evident at rest. Dysarthria, dysphagia and opisthotonus are common. Spasticity, hyperreflexia and extensor plantar reflex appear later. Patients usually show mild to moderate intellectual deficit. Obsessive-compulsive self-mutilation (lip biting or finger chewing) may appear as soon as teeth are present, does not result from lack of sensation, and may be associated with or aggravated by psychological stress. Aggressive behavior (i.e. spitting, abusive language) may be directed against family and friends. Megaloblastic anemia is common and may be severe. Microcytic anemia may occur. UAO can lead to joint inflammation, gouty arthritis, and urolithiasis. Renal failure or acidosis occurs rarely. Patients may die from aspiration pneumonia or complications from chronic nephrolithiasis and renal failure. With optimal care, few patients live beyond 40 years and most are confined to a wheelchair.

4.9. i) What is meant by a "***clinical trial***"? ii) What phases does each phase of a "clinical trial" consist of and what is its purpose? iii) The National Institutes of Health (NIH) portal https://www.clinicaltrials.gov is a database that collects clinical trial studies from all over the world. Search this portal for clinical trials directed against enzymes involved in oncometabolite production. Download the information obtained, include it in the report and comment on it.

Group 7 (Biochemistry Grade) response: *i) A clinical trial is an experimental evaluation of a product, substance, drug, diagnostic or therapeutic technique that, in its application to human beings, aims to assess its efficacy and safety. It is only performed when there is reason to believe that the treatment being studied may be beneficial to the patient.*

ii) Phase I. This is the first step in the investigation of a new substance or drug in humans. These are pharmacokinetic and pharmacodynamic studies that provide preliminary information on the effect and safety of the product in healthy subjects or, in some cases, in patients, and will guide the most appropriate administration pattern for subsequent trials.

Phase II. This represents the second stage in the evaluation of a new substance or drug in humans. It is performed in patients suffering from the disease or clinical entity of interest. Its purpose is to provide preliminary information on the efficacy of the product, to establish its dose-response relationship, to know the variables used to measure efficacy and to expand the safety data obtained in Phase I. In general, these clinical trials will be controlled and with randomized treatment assignment.

Phase III. These are trials designed to evaluate the efficacy and safety of the experimental treatment by trying to reproduce the usual conditions of use and considering the therapeutic alternatives available in the indication studied. It is carried out with a larger sample of patients than in the previous phase and is representative of the general population for which the drug is intended. These studies will preferably be controlled and randomized.

Phase IV. These are clinical trials conducted with a drug after it has been marketed. These trials may be similar to those described in phases I, II, III if they study some aspect that has not yet been evaluated or conditions of use different from those authorized, such as a new indication. These studies will preferably be controlled and randomized.

iii) To perform the searches we entered in "Other terms" the names of the three enzymes involved in the production of oncometabolites, either together or separately. In addition, we focused the search on cancer or tumors.

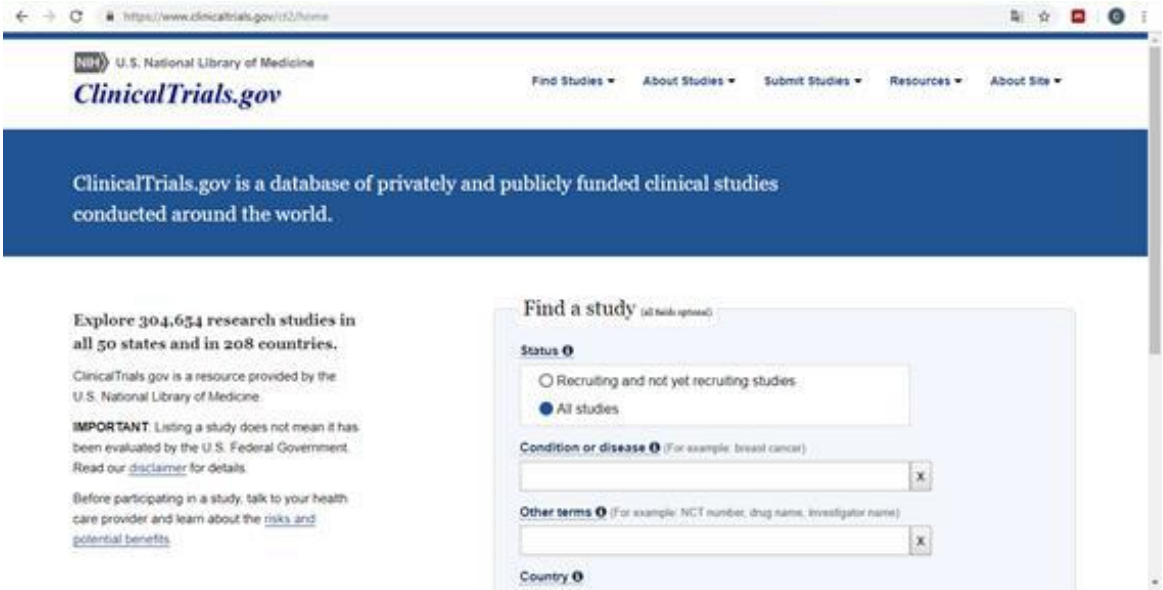

The information obtained from the web is included in the appendix, and consists of three tables, one for each enzyme that has been searched, in which the following information is collected:

- The title of the study.

- The status of the study.

- Whether results are available or not.

- The conditions studied.

- The interventions.

- The locations where the trial is being conducted.

Studies can be found in different statuses; in the information we have obtained we found trials in the following statuses:

- "Active, not recruiting": the study is ongoing, but no new participants are being sought.

- Recruiting": participants are being sought to conduct the trial.

- "Not yet recruiting": has not yet started recruiting patients.

- "Unknown status": at least two years have passed since the trial status was updated.

- Completed": the trial has ended.

Only one of the clinical trials found studies patients with defects in the three enzymes we are studying; however, this trial is not directed against any of these enzymes, but against glutaminase. This trial is entitled "Study of the Glutaminase Inhibitor CB-839 in Solid Tumors". This is an active trial in which no participants are being sought, with no results yet available, conducted by research centers in different states of the United States. This trial is designed for about 205 participants and is expected to be completed in September of this year.

This is a Phase 1 trial evaluating the effect of the selective glutaminase inhibitor CB-839. The trial is being conducted in patients with advanced solid tumors and in patients with triple negative breast cancer, adenocarcinoma, renal cell cancer, mesothelioma or tumors associated with defects in FH, SDH or IDH.

The study entitled "Hereditary Leiomyomatosis Renal Cell Cancer - Study of the Genetic Cause and the Predisposition to Renal Cancer" is an open-label trial, in which participants are still being

sought, conducted by the National Institutes of Health Clinical Center in Maryland, USA. This trial is based on research into the causes of hereditary renal leiomyomatosis and hereditary cell cancer (HLRCC), and how this disease is related to the development of renal tumors. This disease is caused by mutations in fumarate hydratase, and one of the aims of the study is to determine the incidence and characteristics of fumarate hydratase mutations associated with HLRCC.

The trial entitled "Olaparib and Ceralasertib in Treating Patients With IDH Mutant Cholangiocarcinoma or Solid Tumors" is a phase 2 trial not yet seeking participants and with no results available, in which they are studying how two drugs (olaparib and cerelasertib) work in patients with solid tumors or cholangiocarcinoma with mutant IDH. These drugs can stop the growth of tumor cells by blocking some of the enzymes necessary for cell growth. The trial is estimated to be completed in May 2020.

A study also targeting patients with mutant IDH is Phase I Study of BAY1436032 in IDH1-mutant Advanced Solid Tumors, an active, non-patient seeking trial with no results available and conducted jointly by the United States and Germany. This trial aims to determine the safety, tolerability and maximum recommended dose of BAY 1436032 in patients with advanced tumors and IDH1 mutations. Completion of this trial is scheduled for June of this year.

Another of the multiple studies aimed at patients with IDH mutated solid tumors that we are going to discuss is the trial entitled "Metformin And Chloroquine in IDH1/2-mutated Solid Tumors". This study carried out in different centers in the Netherlands is in an unknown status and no results are available. The aim of the trial is to evaluate the toxicity and efficiency of two drugs, metformin and chloroquine, in patients with glioma, intrahepatic cholangiocarcinoma or chondrosarcoma with IDH 1/2 mutated. This trial was scheduled to end in December 2016, but the unknown status tells us that information about the trial has not been updated, so we cannot know whether the trial ended or what its results were.

Finally, we would like to comment on a completed trial with results, the "Linsitinib in Treating Patients With Gastrointestinal Stromal Tumors" study. This study, carried out by several centers in the United States, evaluated the performance of linsitinib in young and adult patients with gastrointestinal tumors. It analyzed drug response, duration of response, adverse effects, serum protein expression patterns, the number of participants who had a metabolic response to the drug and changes in tumor metabolism, among other aspects. The study was completed in 2015. The study results include a summary of the participants' progress at each stage of the study, information collected on all participants prior to the start of the trial, measures taken to determine the effect of the drug as indicated by the trial protocol, and possible side effects that could occur.
