# Supplementary material for: Turning around Cycles: An Approach Based on Selected Problems/Cases to Stimulate Collaborative Learning about Krebs and His Four Metabolic Cycles
Source: J Chem Educ. 2022 May 25;99(6):2270–6. doi: 10.1021/acs.jchemed.1c01038 (PMC9202563; doi:10.1021/acs.jchemed.1c01038)
Supplement: Supplementary file 6 — ed1c01038_si_006.pdf [file ed1c01038_si_006.pdf]

PERCEPTION TEST ON KREBS AND HIS FOUR METABOLIC CYCLES PBL

17. After taking this course, what does Problem Based Learning mean to you?

Nickname: *Nickname: «To take part in this PBL means to have a greater involvement in the Metabolic Regulation course. You can reinforce the acquired knowledge and you learn to work in grouop within a limited period of time».*

Nickname: *Unicorn: «I think this a good learning method and a good way to approach the subject, but I fell that I needed more help from professors».*

Nickname: *Darth Vader: «It has been something new, which has made me to be more involved in the course, although this has taken more time and effort».*

Nickname: *NADH: «I think it is a convenient and very useful approach to the contents of the subject. I feel that I am going to be better prepared for the exam. However, my impression is that this makes student to work more and not always it is easy to combine it with the efforts devoted for the rest of subjects».*

Nickname: *Kiro: «I think this has been a very novel activity to settle knowledge on topics that had not been explored in depth in the regular classroom».*

Nickname: *R1977: «I understand this activity as the use of problems and cases, either real or not, to trigger the need to search autonomously information to learn or to settle knowledge in an alternative and complementary wat to "normal" classes».*

Nickname: *R1975: «To solve problems and cases in small groups can make it easier to achieve an acceptable response. Furthermore, each one can observe how the rest of the members of the group works and this contributes to enhance the skill to work in teams ».*

Nickname: *Romeo: «(The experience) has meant to learn contents of the subject in a more independent way, but always counting with the professor's guidance, who proposed to us a number of exercises and problems interesting and relevant».*

.
